# Chitosan Nanoparticles Co-Encapsulating Selegiline Analogue and L-Tyrosine Mitigate Depression-Related Pathology and Cognitive Decline in Rats

**DOI:** 10.3390/biom16060871

**Published:** 2026-06-14

**Authors:** Wesam Abd El-Fattah, Ahlem Guesmi, Naoufel Ben Hamadi, Khulud M. Alshehri, Ehab Mohamed Abdella, Rehab R. Mohamed, Reda F. M. Elshaarawy, Hani S. Hafez

**Affiliations:** 1 Department of Chemistry, College of Science, Imam Mohammad Ibn Saud Islamic University (IMSIU), P.O. Box 5701, Riyadh 11623, Saudi Arabia; wabdulfatah@imamu.edu.sa (W.A.E.-F.); nabenhamadi@imamu.edu.sa (N.B.H.); 2 Department of Biology, Al-Baha University, Al Baha 65431, Saudi Arabia; kalshehri@bu.edu.sa; 3 Zoology Department, Faculty of Science, Beni Suef University, Beni-Suef 62521, Egypt; immohammad@bu.edu.sa; 4 Biology Department, Faculty of Science, Al-Baha University, Al-Baha 65779, Saudi Arabia; 5 Zoology Department, Science Faculty, Fayoum University, Fayoum 63514, Egypt; rrm01@fayoum.edu.eg; 6 Department of Chemistry, Faculty of Science, Suez University, Suez 43533, Egypt; 7 Department of Zoology, Faculty of Science, Suez University, Suez 43533, Egypt

**Keywords:** chronic unpredictable stress depression-like, cognitive dysfunction, monoamine oxidase inhibitors, oxidative stress, neuroinflammation, nano drug delivery

## Abstract

Chronic depression is associated with oxidative stress, neuroinflammation, neurotransmitter imbalance, and Alzheimer’s-like changes. Current monoamine oxidase inhibitors have limited cognitive benefits and disease-modifying properties. A new nanotherapeutic, combining chitosan nanoparticles, propargylamino-1-(4-methylthiophenyl) propane (PAMTP), and L-tyrosine (En@PAMTP_Tyr), was developed. En@PAMTP_Tyr nanoparticles were ~140 nm in diameter, with a zeta potential of +27 mV and entrapment efficiencies of 73.45% for PAMTP and 90.85% for L-tyrosine. Drug release was pH-sensitive, favoring acidity. Intraperitoneal administration of En@PAMTP_Tyr reduced anhedonia, despair, cognitive deficits, and neuromuscular weakness, with efficacy matching or exceeding that of selegiline. In treated rats’ hippocampal tissue, En@PAMTP_Tyr increased superoxide dismutase and glutathione, normalized MAO and acetylcholinesterase activities, and corrected CUSD-induced TNF-α and IL-10 changes, showing antioxidant and anti-inflammatory effects. Histological analyses revealed that En@PAMTP_Tyr preserved CA1 pyramidal neurons, reduced β-amyloid levels, restored tau protein, and improved brain-derived neurotrophic factor levels, indicating reduced neurodegeneration. Molecular docking studies showed that PAMTP had high affinity for monoamine oxidase and acetylcholinesterase, supporting its role as an MAO-B inhibitor and cholinergic modulator. These findings suggest that En@PAMTP_Tyr is a promising nanoplatform for targeting MAO-B in depression, addressing mood, cognitive function, oxidative stress, inflammation, and Alzheimer-like pathology in the hippocampus.

## 1. Introduction

Depression causes persistent cognitive impairment and affects approximately 300 million individuals worldwide. This condition leads to irritability, sleep disturbances, fatigue, guilt, hopelessness, concentration difficulties, and suicidal thoughts [1]. Therefore, the chronic unpredictable stress (CUS) model investigates depression by subjecting animals to random stressors, such as food and water deprivation, which disrupt homeostasis and induce depressive behaviors [2]. Current antidepressant treatments have significant limitations, as patients often fail to respond adequately, cannot tolerate treatment, or relapse after initial improvement [3].

Depression, a mood disorder, can increase the risk of brain diseases, double the chance of Alzheimer’s disease (AD), and explain 8–11% of dementia cases. Depression and AD share causes long-term stress, harming brain cells, leading to protein build-up and changes, brain inflammation from high chemical levels, and reduced brain growth factors affecting cell growth and connections. This creates a cycle of depression that may accelerate AD-like brain changes, worsening depression by causing brain cell loss and chemical issues [4].

Moreover, traditional antidepressants that target monoamine systems take weeks to exert their full effects, leaving patients vulnerable to distress and increasing suicide risk [5]. The compound 2-N-Propargylamino-1-(4-methylthiophenyl) propane (PAMTP) contains a propargyl group linked to a propane chain and a 4-methylthiophenyl group, enhancing its lipophilic properties for central nervous system (CNS) interactions by influencing neurotransmitter levels, such as serotonin, and potentially affecting mood disorders, as compounds like selegiline show antidepressant effects [6]. PAMTP replaces the N-methyl group of selegiline with a 4-methylthiophenyl group on the propane backbone, possibly increasing lipophilicity and CNS penetration while maintaining propargylamine binding to MAO-B [7]. Tyrosine is key for mood-regulating neurotransmitters, such as dopamine and norepinephrine, which are disrupted in depression, and its supplementation shows promise for improving mood during stress or sleep deprivation [8].

Direct clinical trials testing MAO inhibitors with L-tyrosine are limited. Early research shows they might work well together. L-tyrosine reduces stress and improves mood and cognitive function. In animal studies, L-tyrosine in nanoparticle form is more effective than regular L-tyrosine because it better reaches the brain [8]. Selegiline, a prototypical MAO-B inhibitor, exhibits antidepressant and neuroprotective effects (including BDNF modulation) in MDD and PD-associated depression [9]. MAO inhibitors help maintain certain brain chemicals and lower harmful molecules. Adding tyrosine helps correct issues with brain chemicals and their production [10].

Depression and stress-related disorders decrease brain-derived neurotrophic factor (BDNF) levels, reducing neurogenesis, causing cholinergic dysfunction with increased AChE activity, and impairing long-term potentiation (LTP) in the hippocampus, disrupting cortical function. Inhibitors of these factors improve mood and restore memory [11]. Elevated monoamine oxidase (MAO) levels are linked to oxidative pathways that produce reactive oxygen species (ROS) [12]. Selegiline, known for its neuroprotective effects, acts as a selective MAO-B inhibitor, elevating brain dopamine levels. However, it loses selectivity at higher doses, limiting its use due to side effects [13].

Depression extends beyond mood disruption to affect neurology more broadly, with growing research exploring its link to dementia risk, particularly Alzheimer’s disease. Findings operate at two levels: some studies connect a formal depression diagnosis to AD risk, while others find associations with depressive symptoms even without a formal diagnosis. Sex differences also emerge, with AD risk tied to depression appearing more prominently in men, while depressive symptoms more strongly impact cognitive function in women [14]. Depression may serve as an early indicator of AD, manifesting before cognitive symptoms; this theory is supported by Dal Forno et al. [15] and Stewart [16]. This neurological challenge underscores the novelty and importance of developing therapeutic strategies with that address emotional, cognitive, and neuroprotective factors. Moreover, implementing a strategy that targets multiple domains is hindered by pharmacokinetic limitations. En@PAMTP_Tyr addresses these challenges, particularly the BBB impermeability and low oral bioavailability that limit central nervous system (CNS) efficacy [17]. PAMTP, an improved selegiline analog, shows better CNS penetration but remains vulnerable to peripheral metabolic degradation, reducing brain delivery and increasing systemic side effects [18]. L-tyrosine, a catecholamine precursor, also faces challenges: its hydrophilic nature limits BBB traversal and CNS availability [19]. These challenges necessitate the development of an encapsulation strategy to transport both agents effectively to the brain.

Nanoparticle encapsulation directly addresses these permeability challenges by providing a physicochemical shield that protects labile payloads from premature metabolic degradation, facilitates transcytosis across the BBB, and enables controlled release within the CNS. In this study, the chitosan nanoparticle formulation (En@PAMTP_Tyr) administered intraperitoneally benefited by (i) bypassing first-pass metabolism for higher bioavailability, (ii) pH-responsive release of PAMTP and L-tyrosine, (iii) enhanced BBB crossing via the cationic matrix, and (iv) protection from degradation. These features led to rapid improvements in depressive behaviors, cognitive function, oxidative stress, neuroinflammation, and hippocampal neuroprotection, with effects equal to or superior to those of selegiline. Future development could adapt this platform to injectable or intranasal routes for fast-onset effects [19]. This ensures co-delivery and controlled, pH-responsive release at the target site. Intraperitoneal (IP) administration bypasses first-pass metabolism, ensuring high bioavailability and rapid action, unlike oral antidepressants [17]. This method is supported by studies on injectable nanoformulations of ketamine and lithium carbonate for depression [19], validating this route for MAOI-based therapies.

The synergistic effect stems from complementary catecholaminergic neurotransmission mechanisms. Selegiline analog with a propargylamine group (PAMTP) irreversibly inhibits monoamine oxidase B (MAO-B), reducing dopamine degradation, preserving synaptic monoamine levels, and decreasing hydrogen peroxide from MAO-mediated oxidative deamination. However, MAO-B inhibition alone does not address dopamine precursor depletion from chronic stress or directly enhance catecholamine synthesis when substrate is limited [20]. L-tyrosine is a precursor to L-DOPA, dopamine, norepinephrine, and epinephrine. In stress-induced depression, L-tyrosine supplementation restores the neurotransmitter synthesis substrate pool, supporting catecholaminergic tone. Combining MAO-B inhibition (preventing monoamine degradation) and tyrosine supplementation (replenishing substrate) reduces monoamine catabolism and increases anabolism. Literature supports MAO inhibitor–amino acid precursor synergy, and animal studies show nanoformulated tyrosine is more effective than its free form [21].

The novelty of this work is tripartite: (i) PAMTP represents a structurally differentiated selegiline analog bearing a 4-methylthiophenyl substitution on the propane backbone, which is predicted to confer greater lipophilicity and CNS penetration relative to selegiline; (ii) the chitosan nanoparticle delivery system overcomes the well-documented pharmacokinetic limitations of both PAMTP and L-tyrosine individually, achieving pH-responsive release, high entrapment efficiency, and facilitated BBB crossing; and (iii) their synergistic co-encapsulation constitutes a rationally designed dual-payload nanotherapeutic that addresses depression not as a monoamine deficit alone, but as a convergent pathology involving oxidative stress, neuroinflammation, cholinergic dysregulation, and Alzheimer-like hippocampal changes.

## 2. Materials and Methods

### 2.1. Materials

Details regarding the chemicals and reagents employed in this study are presented in the Electronic Supplementary Material (ESM). The protocols utilized for the preparation of 2-N-propargylamino-1-(4-methylthiophenyl) propane (PAMTP), a selegiline mimic, are described in the ESM. Highly deacetylated chitosan (HDCS) was obtained as described in a previous study by Mohamed et al. [22]. The methodologies and instruments used for the characterization of PPE, newly synthesized materials, and their precursors are documented in the ESM.

### 2.2. Synthesis of 2-N-Propargylamino-1-(4-methylthiophenyl)propane (PAMTP)

PAMTP was synthesized from 1-(4-methylthiophenyl)-acetone (3) and propargylamine HCl following a previously described procedure [23], with slight alterations. Briefly, a stirred solution of compound (3) (11.45 mmol) in dry methanol (50 mL) was combined with propargylamine HCl (80 mmol) and sodium cyanoborohydride (15.92 mmol, 1.00 g), and the mixture was stirred at 20 °C for 72 h. The pH was periodically adjusted to 5–6 using 4 M methanolic HCl, as indicated by damp universal pH paper. Excess hydride was neutralized by adding 10% aqueous HCl (150 mL), and the resulting aqueous layer was washed with dichloromethane (3 × 50 mL). The aqueous layer was then made basic with 15% aqueous NaOH solution and extracted with dichloromethane (3 × 50 mL). The combined organic layers were dried over anhydrous Na_2_SO_4_, and the volatiles were removed under reduced pressure, yielding the product as a colorless oil (33%). The spectral (ATR-IR, NMR) (Figures S1 and S2) and other characterization data of PAMTP are provided in the ESM.

### 2.3. Preparation of Encapsulated PAMTP/Tyr-Loaded Chitosan Nanoparticles (En@PAMTP_Tyr)

Chitosan nanoparticles co-encapsulating PAMTP and L-tyrosine (En@PAMTP_Tyr) were prepared by a modified ionotropic gelation process following the method described by Patel and Jain [24], with minor modifications. Chitosan powder (0.4 g, MW = 6200 kDa, degree of deacetylation (DDA) = 96.1%) was added to a 250 mL round-bottom flask containing 100 mL of 1 wt% acetic acid. The mixture was sonicated (40 kHz, 250 W) for 15 min at room temperature (25 ± 1 °C) and stirred (400 rpm) at 60 °C for 4 h to obtain a clear chitosan stock solution. The prepared solutions of Tyr and PAMTP (0.3 g each in 30 mL of distilled water and ethanol, respectively) were added to the CS stock solution progressively and concurrently while being vigorously stirred (600 rpm) for two hours to form a homogeneous drug–polymer dispersion. To obtain En@PAMTP_Tyr nanoparticles (nanoformulation), sodium tripolyphosphate (TPP) (0.03% *w*/*v*) was subsequently introduced incrementally as a polyanionic crosslinker into the PAMTP/Tyr/HDCS mixture to achieve a chitosan:TPP mass ratio of 5:1, under continuous stirring (300 rpm) for 24 h at room temperature. This ratio was determined based on preliminary optimization and reported literature [25]. After stirring for 24 h under ambient conditions, the synthesized En@PAMTP_Tyr nanoformulation product was collected by centrifugation at 12,000 rpm for 30 min and thoroughly rinsed with distilled water. The obtained En@PAMTP_Tyr was freeze-dried at −80 °C for 24 h and stored at −20 °C in a sealed vial for later use, to preserve their physicochemical stability and prevent moisture uptake.

### 2.4. Loading Capacity and Encapsulation Efficiency

Based on the prior research of Jafari et al. [26], we used UV-Vis spectrophotometry to calculate the encapsulation efficiency (EE) and loading capacity (LC) of PAMTP and Tyr. The aqueous sample dispersion (100 μL, 10 mg/mL) was boiled with a 1 M solution of HCl at 95 °C for 30 min to lyse the sample and liberate the encapsulated PAMTP and Tyr. Subsequently, 2 mL of ethanol was added after cooling, and the mixture was centrifuged at 10,000 rpm for five min at room temperature. The liberated PAMTP and Tyr were measured spectrophotometrically at 259 and 275 nm, respectively. Equations (1) and (2) were used to determine the *LC* and *EE* of the drug.

(1) $$LC\%=\frac{Initial\;drug-free\;drug}{W_{Nanoformulation}}\times 100\;$$

(2) $$EE\%=\frac{Initial\;drug-free\;drug}{Initial\;drug}\times 100\;$$

Notably, individual UV–Vis absorption profiles of PAMTP and L-tyrosine were separately analyzed. As for the UV visible region, both compounds produced partially overlapping bands with PAMTP displaying a significant band around λmax of ∼259 nm and Tyr at about 275 nm, causing spectral interference in solutions containing both compounds. The overlap suggests that single-wavelength simple spectrophotometry alone is inadequate to discriminate and quantify each drug in the dual-payload En@PAMTP_Tyr system, unless further complex deconvolution or separation protocols are applied. To show details, the measured spectra of PAMTP and Tyr and their overlaid profiles are listed in ESM (Figures S3 and S4).

To confirm that the harsh hydrolytic conditions employed during encapsulation-efficiency assessments did not actively degrade the analyte, we compared the UV–Vis spectra of free L-tyrosine and PAMTP following exposure to 1 M HCl at 95 °C for 30 min. The post-treatment spectra for both compounds confirmed that the λmax positions (and overall band shapes) of untreated solutions were retained, with only minor absorbance intensity differences (<5%), confirming negligible degradation at these concentrations and validating that this protocol is sufficient to measure quantitative nanoparticle lysis. The validation spectra are now supplied in the ESM (Figures S5 and S6).

While spectral overlap between PAMTP and Tyr introduces quantification uncertainty, measurements at 259 nm and 275 nm primarily reflect the absorbance of PAMTP and Tyr, respectively, allowing semi-quantitative estimation of individual drug content. Future studies employing HPLC or multivariate calibration methods would provide more precise simultaneous quantification.

### 2.5. In Vitro Release

The in vitro release of PAMTP and Tyr from the En@PAMTP_Tyr nanoformulation, was determined under acetate buffer (AB, 5.5 pH) and phosphate-buffered saline (PBS, 7.4 pH) to mimic mild acidic conditions and physiologic environments respectively. Briefly, En@PAMTP_Tyr (50 mg) was placed in 10 mL of the corresponding release medium and then incubated at 37 ± 0.5 °C under agitation with a magnetic stirrer (100 rpm) to continuously maintain the suspension homogeneous. At scheduled release times, 2 mL samples were taken from the release medium and centrifuged at 10,000 rpm for 5 min to separate any suspended nanoparticles before spectrophotometric analysis. The clear supernatant was subsequently analyzed using a UV-Vis spectrophotometer at 259 nm and 275 nm for PAMTP and Tyr, respectively. After each sampling, the withdrawn volume was immediately replaced by an equal volume of fresh prewarmed medium that retained the same temperature, to preserve constant volume and sink conditions. The cumulative release percentage (R%) of PAMTP and Tyr was calculated according to the following formula (Equation (3)):

(3) $$R\%=\frac{released\;drug\;at\;time\;t}{Initial\;drug\;in\;Nanoformulation}\;\times 100\;$$

### 2.6. Biological Studies

#### 2.6.1. Depression Model Induction, Experimental Procedures, and Treatments

This study used 50 adult male albino rats, each weighing 180–220 g and aged 8–10 weeks, from the National Research Centre in Giza, Egypt. Rats were housed in 50 × 40 × 20 cm cages under a 12 h light-dark cycle at 27 °C and 60–65% humidity. During the first week, rats received a standard diet and water. Chronic unpredictable stress (CUS), or mild stress (CUMS), is a rodent depression model replicating human stressors like food and water deprivation. The study followed Zeldetz et al. [2] protocol with modifications to induce depression-like behavior in rats via daily random stressors over five weeks, one by day, another by night, to maximize unpredictability and prevent habituation. Stressors included 18 h food and water deprivation, exposure to empty bottles post-deprivation, and other mild stressors (e.g., cage tilting, damp bedding, altered light/dark cycles, social isolation, restraint stress, overnight illumination), aligning with standard CUS/CUMS protocols. This regimen disrupts homeostasis without physical harm, reliably inducing depression-like states. After five weeks, the rats were returned to standard conditions with ad libitum food and water for two weeks before behavioral testing. Initially, 50 rats were divided into five groups (see Figure 1): (1) Control: healthy untreated animals; (2) En@PAMTP_Tyr: healthy animals receiving the nanoformulation; (3) CUSD: chronic unpredictable stress depression untreated animals (positive control for depression); (4) CUSD + En@PAMTP_Tyr: chronic unpredictable stress depression animals treated with the nanoformulation at 6 mg/kg/day; and (5) CUSD + Selegiline: depression-induced animals treated with free selegiline 6 mg/kg/day [27]. After five weeks of CUSD induction, rats were removed from stress conditions and kept under standard conditions with free food and water for two weeks. All groups then underwent behavioral assessments, including the sucrose preference test for anhedonia, tail suspension test for despair-like behavior, novel object recognition test for cognitive function, Passive Avoidance test for learning and memory, and grip strength test. This allowed analysis of chronic stress effects and treatment efficacy in mitigating depression-like symptoms, providing insights into therapeutic strategies for stress-induced depression, as shown in Scheme 1. All animals were treated according to laboratory guidelines. ARRIVE 2.0 guidelines were followed for handling animals [28]. The study was conducted following the Declaration of Helsinki, approved by the Institutional Review Board of Sinai UNIVERSITY (protocol code Approval No.: SU_REC 2025 (105A)) for studies involving Animals.

#### 2.6.2. Animal Behavioural Tests

The behavioral battery was designed to capture the multidimensional clinical presentation of major depressive disorder across affective, cognitive, and neuromuscular domains, consistent with the study’s translational scope, as follows:

##### Sucrose Preference Test

The sucrose preference test was selected as the primary measure of anhedonia—the reduced capacity to experience pleasure-a core affective symptom of depression and a reliable readout of the CUSD model. The test was administered at 9:00 a.m. Rats were housed individually in two bottles containing 100 mL of sucrose solution (1%, *w*/*v*) and water. After 4 h, the volumes of sucrose and water consumed were recorded. The sucrose preference was calculated using formula (Equation (4)) according to Huang et al. [29]:

(4) $$Sucrose\;Preference\left(\%\right)=\frac{sucrose\;consumption\left(ml\right)}{sucrose\;consumption\left(mL\right)+water\;consumption\left(mL\right)}\;\times \;100$$

##### Novel Object Recognition (NOR) Test

The novel object recognition test assessed hippocampus-dependent recognition memory and cognitive flexibility, both impaired in depression and relevant to the study’s focus on hippocampal neuroprotection. The test included training and test sessions, with a retention time of 60 min. Each rat was individually housed before training. During training, two identical plastic objects (A and B) were similar in size with the same color (6–7 cm) and symmetrically glued 8 cm apart on a plexiglass board (5 × 15 cm). Each animal was allowed to explore the objects for 3 min. Exploration was defined as the rat facing, touching, or sniffing an object. The total exploration time for each object was recorded by a blinded observer. The board was removed after training. During retention, one identical and one novel object (A and C) was used, and the exploration time was recorded for 3 min (Figure 1) [30]. Because absolute exploration times varied between animals, times were normalized [(time exploring object A) + (time exploring object B/C) = 100%] and expressed as a percentage of total exploration. Increased novel object exploration indicates successful memory retention.

(5) $$Exploration\;times(\%)=\frac{time\;exploring\;object\;C-time\;exploring\;object\;A/B}{time\;exploring\;object\;C+time\;exploringobject\;A/B}\times 100\;$$

##### Passive Avoidance Test

The passive avoidance test measures aversive memory consolidation and retrieval, probing a distinct hippocampal memory circuit and offering additional resolution of the cognitive phenotype. A shuttle box with two compartments (illuminated and dark, 20 × 20 × 30 cm) separated by a sliding door was used to study passive avoidance learning and memory. Both compartments had stainless-steel bar floors with an isolated stimulator delivering electrical shocks to the dark chamber. The equipment included a white-lighted section and dark compartment divided by a downward-opening door (25 cm × 11 cm), as shown in Figure 1 (2 PAT). During acquisition, rats were placed in a light room to explore and received a 0.5 mA shock for 5s in the dark compartment. After the door was reopened, the rats returned to the lightroom. The entry time into the dark chamber was recorded. Rats were disqualified if they stayed in the light compartment for over 180s or did not enter the dark section. For short-term memory assessment, a retention test was performed 30 min post-acquisition, measuring dark section entry time as retention latency (maximum 180s) [31].

##### Grip Strength

The grip strength test assessed the neuromuscular domain, given depression’s association with reduced physical performance and muscle weakness, and the link between dopaminergic and cholinergic signalling and neuromuscular function. The grip strength of the rats was assessed based on their natural inclination to grasp when pulled backward. A horizontal bar with a force transducer was used to evaluate the forelimb grip strength. The rat was held by its tail with its front paws above the grip bar and then pulled backward until it grasped the bar with both forepaws. The maximum tension was recorded using a digital force gauge upon release. The results are expressed as multiples of body weight. A dynamometer was used to measure the force (Figure 1). This was repeated two to three times for each hand, and the highest score was recorded [32].

##### Tail Suspension

The tail suspension test (TST) was included as an established measure of behavioural despair and passive coping, providing a complementary index of depressive-like affect independent of reward processing. The test evaluates potential antidepressant drugs by assessing behaviours indicative of despair, although a version for rats is also available. In this test, the rat is hung by its tail for approximately 6 min, during which it alternates between intense activity (struggling to escape) and stillness (passively hanging with little movement). The main focus of the test is the estimation of the total time spent immobile, as antidepressants such as selegiline usually decrease this duration, suggesting antidepressant-like properties [27].

#### 2.6.3. Tissue Preparation

##### Transcardial Perfusion and Fixation

After habituation for 1 h post-experiments, the rats were anesthetized with intraperitoneal pentobarbital (30 mg/kg), isoflurane, and 30 mL PBS (pH 7.4), followed by brain and hippocampal dissection on ice. One half was stored in RNAlater at −80 °C for antioxidant, cytokine, and enzyme analyses, and the other half in 10% formalin for paraffin-embedded coronal sectioning (5 µm slices) for staining and immunohistochemistry.

##### Histological and Immune-Histochemical Examination of the Hippocampus Complex

Paraffin sections were mounted and deparaffinized using xylene and a descending series of alcohol concentrations (absolute, 90, 70, 50, and 30%). Hematoxylin and eosin (H&E) staining was performed following the protocol established by Bancroft and Gamble [33]. For immunohistochemical examination, sections were mounted on positively charged glass slides and treated with 0.3% H_2_O_2_ in PBS for 30 min, followed by 10% goat serum in PBST for 30 min. Brain tissue slices were cleaned with xylene and rehydrated in alcohol after paraffin removal during a 30 min incubation at 60 °C. Additionally, a 10% H_2_O_2_ solution with methanol was applied for 10 min to inhibit endogenous peroxidase activity. The sections were rinsed with Tris-buffer solution, and antigen retrieval was conducted using citrate buffer for 8 min. Coronal paraffin-embedded brain sections were incubated overnight with primary antibodies (1:200) targeting tau protein (Cat# A1103, AB clonal Technology, Woburn, MA, USA), β-amyloid (Cat#A17911), and BDNF (Cat# A4873), followed by a two-hour incubation with a rabbit polyclonal secondary antibody. DAB (Sigma, Ronkonkoma, NY, USA) was utilized, and hematoxylin was employed as a counterstain for microscopic and immunoreactivity assessment [34].

#### 2.6.4. Antioxidant Assessments

##### Superoxide Dismutase (SOD) Activity

Superoxide dismutase (SOD) activity was measured using the spectrophotometric method described by Kakkar et al. [35]. The reaction mixture consisted of 1.2 mL of sodium pyrophosphate buffer (0.052 M, pH 7.0), 0.1 mL of phenazine methosulfate (186 μM), and 0.3 mL of the tissue supernatant, obtained by sequential centrifugation of the hippocampal homogenate at 1500× *g* for 10 min followed by 10,000× *g* for 15 min at 4 °C. The enzymatic reaction was initiated by adding 0.2 mL NADH (780 μM) and terminated after 1 min by the addition of 1.0 mL glacial acetic acid. The reaction mixture was mixed with 4.0 mL of n-butanol and centrifuged, and the chromogen-containing butanol phase was measured spectrophotometrically at 560 nm. Enzyme activity was expressed as units per milligram of protein.

##### Glutathione (GSH) Activity

Glutathione (GSH) activity in hippocampal tissue was determined by the method described by Tipple and Rodgers [36], as follows: This colorimetric assay is based on the reaction of free thiol (-SH) groups in GSH with 5,5′-dithiobis-(2-nitrobenzoic acid) (DTNB), yielding yellow 5-thio-2-nitrobenzoic acid (TNB), measurable at 412 nm. Briefly, hippocampal tissue was homogenized in 0.02 M EDTA or ice-cold phosphate buffer (0.1 M, pH 7.4). A 0.5 mL aliquot of the homogenate was mixed with 0.5 mL of 4% sulfosalicylic acid to precipitate proteins. The mixture was centrifuged at 1200× *g* for 10 min at 4 °C. To 0.5 mL of the resulting supernatant, 2.0 mL of 0.3 M phosphate buffer (pH 7.4), and 0.5 mL of DTNB solution (0.01 M in 1% sodium citrate) were added. The absorbance of the yellow chromogen was measured at 412 nm using UV-visible spectrophotometry. The GSH concentration was determined by comparing the sample absorbance values to a standard curve prepared with known concentrations of reduced GSH.

##### Acetylcholinesterase (AChE) Activity

Acetylcholinesterase (AChE) activity in hippocampal tissue was measured using Ellman et al.’s colorimetric method (1961), modified for microplate analysis (Lane et al., [37]. This involves acetylthiocholine iodide (ATCI) hydrolysis to thiocholine, which reacts with 5,5′-dithiobis-(2-nitrobenzoic acid) (DTNB) to form a yellow chromophore at 405 nm. A 96-well plate reaction mixture included 25 μL 15 mM ATCI, 75 μL 3 mM DTNB, and 75 μL 50 mM Tris–HCl buffer (pH 8.0) with 0.1% BSA. After 5 min at room temperature, baseline absorbance at 405 nm was recorded to account for spontaneous hydrolysis. Subsequently, 25 μL of the tissue sample was added and incubated for 5 min. Absorbance was measured at 405 nm, and AChE activity was calculated as μmol/min/g of tissue protein (TP). Samples were assayed in duplicate and triplicate to ensure accuracy.

##### Monoamine Oxidase (MAO) Activity

Monoamine oxidase (MAO) activity was assessed in homogenized tissue using the fluorometric method originally described by Köchli and Von Wartburg [38] and later refined by Youdim and Holzbauer [39]. This method employs kynuramine, a non-selective substrate oxidized by MAO-A and MAO-B isoforms, yielding an unstable aldehyde that cyclizes into 4-hydroxyquinoline, a fluorescent compound that can be detected by a spectrofluorometer. Hippocampal homogenate was prepared in distilled water (5 mg/mL) using a Polytron homogenizer at 4 °C. For the reaction, 1.0 mL of tissue homogenate was incubated with 0.5 mL of 0.5 M phosphate buffer (pH 7.4) and 0.5 mL of kynuramine (200 µg/mL), and the final volume was adjusted to 3.0 mL. After 60 min incubation at 37 °C, the reaction was stopped with 2.0 mL of 10% trichloroacetic acid and centrifuged at 3000× *g* for 10 min. The supernatant (1.0 mL) was mixed with 1 N NaOH (2.0 mL), and fluorescence was measured at 315 nm excitation and 380 nm emission. MAO activity was expressed as fluorescence intensity units per milligram of protein or as micromoles of 4-hydroxyquinoline produced per hour.

#### 2.6.5. Enzyme-Linked Immunosorbent Assay (ELISA) for TNF-α and IL-10

TNF-α and IL-10 concentrations in rat hippocampal tissue were quantified using sandwich ELISA kits (Cat #88-7346-88 and Cat #88-7106-88, respectively; Thermo Fisher Scientific, Waltham, MA, USA) following the manufacturer’s protocols. This method has been validated in neuroinflammatory studies [40]. Tissue homogenates were prepared in cold PBS (pH 7.4) containing protease inhibitors and centrifuged at 10,000× *g* for 15 min at 4 °C. The supernatants were collected for cytokine analysis. Ninety-six-well microplates pre-coated with monoclonal antibodies specific to rat TNF-α or IL-10 were used for the assays. A standard or sample (100 µL) was added to each well and incubated at room temperature for 2 h. After washing, biotin-conjugated detection antibodies were added, followed by streptavidin-HRP conjugate. Color development was performed using TMB substrate. The reaction was stopped with 1N sulfuric acid, and the absorbance was measured at 450 nm. TNF-α and IL-10 concentrations were determined using a standard curve of recombinant rat cytokines and were expressed as pg/mg of protein.

### 2.7. In Silico Studies

The compound 2-N-Propargylamino-1-(4-methylthiophenyl) propane was evaluated using active-site molecular docking simulations, with selegiline serving as a reference compound. The three-dimensional structures of ligand compounds were obtained from http://pubchem.ncbi.nlm.nih.gov (accessed on 23 June 2025)) in SDF format and converted to PDBQT format using OpenBabel [41]. The receptors, monoamine oxidase (3PO7) and acetylcholinesterase (5FPQ), were obtained from https://www.rcsb.org. Protein structures were verified and energy-minimized using the Swiss-PdbViewer software (version 4.1.0) (SPDBV) [42]. Pre-treatment, including the removal of solvent and co-crystallized ligand molecules, was performed using the Biovia Discovery Studio Visualizer (Version 4.5) [43]. The active sites were identified based on the coordinates of the co-crystallized ligands: monoamine oxidase (3PO7) (x = 27.756, y = 124.960, z = 10.795) and acetylcholinesterase (5FPQ) (x = 31.901, y = 37.344, z = 31.285). The grid boxes were set with (40 × 40 × 40) points and 0.375 Å spacing. Molecular docking simulations were performed using AutoDock 4.2, with the Lamarckian genetic algorithm [44]. From the 50 conformations, the optimal conformer was selected based on the lowest binding energy and minimal Ki and was visualized using PyMol (Version 2.0) and Biovia Discovery Studio Visualizer [45].

The docking protocol was validated through the re-docking of co-crystallized ligands into their corresponding binding cavities with the same grid parameters and search algorithms. For monoamine oxidase (3PO7) a RMSD of 0.82 Å was achieved between the re-docked ligand and the conformation given by that derived from crystal structure data; for acetylcholinesterase (5FPQ) an RMSD of 0.95 Å was found, both below the generally accepted threshold of 2.0 Å used to confirm that experimentally determined binding modes can be reliably reproduced using docking protocols validating their application to PAMTP and selegiline.

### 2.8. Statistical and Image Analyses

The animal’s nose was oriented toward an object at 2 cm, indicating exploration. Animals with less than 10 s of exploration during the sample phase or 30 s during testing were excluded. Individual variations in the total quantity of exploration were considered when calculating the discrimination between objects (Fagiolini et al., 2023) [46]. One-way ANOVA and post hoc Newman–Keuls tests were used to compare groups, and a within-group *t*-test (two-tailed) and Pearson correlation coefficient were employed to evaluate whether individual groups discriminated between items [47]. Digital images obtained from CA1 were examined and quantified using ImageJ software (version 1.2.4) and processed using Prism 7 (GraphPad, San Diego, CA, USA). All data are presented as mean ± standard error of the mean for each protein distribution in the CA1 subregion of the hippocampus of the rat brain.

## 3. Results and Discussions

### 3.1. Synthesis Protocol

The synthetic strategy employed to obtain chitosan-loaded 2-N-propargylamino-1-(4-methylthiophenyl) propane (PAMTP) (En@PAMTP_Tyr) nanoparticles via ionotropic gelation is depicted in Scheme 2. The production of PAMTP begins with the Henry reaction, which transforms 4-methylthiobenzaldehyde (1) into intermediate (2). The subsequent steps involved the reduction and reductive amination of (2) to form the target compound. PAMTP is a selegiline hybrid with a propargylamine moiety that provides neuroprotection, making it a potential medicine for the treatment of Parkinson’s disease. Simultaneously, HDCS is produced by deacetylating CS, which enhances its capacity for ionic interactions and solubility. Finally, L-tyrosine (Tyr) and PAMTP were co-encapsulated into chitosan nanoparticles. This was accomplished using an ionotropic gelation assembly and sodium tripolyphosphate (TPP) as a crosslinker, yielding En@PAMTP_Tyr.

### 3.2. Physicochemical Characterization

Figure 2A shows the FTIR spectra of the encapsulated PAMTP/Tyr-loaded chitosan nanoparticles (En@PAMTP_Tyr) and their precursors, PAMTP, L-tyrosine (Tyr), and HDCS, as well as the final. The HDCS spectrum displays characteristic bands at 3330 cm^−1^ (O–H and N–H stretching) and 2890 cm^−1^ (C–H stretching), and a prominent peak at 1023 cm^−1^ corresponding to C–O–C stretching, indicating the presence of saccharide units [48]. PAMTP shows characteristic peaks at 3260 cm^−1^ (C≡C–H stretching), 3180 cm^−1^ (N–H stretching), 2146 cm^−1^ (C≡C stretching), 1595 cm^−1^ (aromatic C=C), and 1250–1020 cm^−1^ (C–N and C–S stretching), which are consistent with those expected for propargylamine and thioaryl moieties [49]. The amino acid tyrosine shows broad stretches of O–H and N–H groups around 3430 cm^−1^, a sharp C=O stretch near 1680 cm^−1^, and vibrations of the aromatic ring near 1603–1512 cm^−1^ [50].

Upon encapsulation, the FTIR spectrum of En@PAMTP_Tyr exhibited slight but detectable changes in the FTIR spectrum compared with HDCS (Figure 2A), with more obvious differences observed by examining expanded panels (Figure 2B,C). The extended amide I region (1700–1600 cm^−1^) (Figure 2B) disclosed that the HDCS band is shifted from 1655 cm^−1^ to 1645 cm^−1^ after encapsulation amounts indicating electrostatic forces between the protonated amino groups of chitosan and Tyr with carboxylate group. Similarly, In the 3500–3100 cm^−1^ region (Figure 2C), the broad O–H/N–H stretching band was centered at 3330 cm^−1^ for HDCS; however, this peak appeared somewhat broader and shifted to 3320 cm^−1^ after encapsulation indicating enhanced H-bonding interactions arising from chitosan hydroxyl and amino groups and functional groups introduced during loading PAMTP and Tyr. In addition, the shift in the propargyl group peak from 2146 cm^−1^ in the PAMTP spectrum to 2132 cm ^−1^ in the En@PAMTP_Tyr spectrum indicates that PAMTP was successfully encapsulated and that the propargyl group was involved in binding to the chitosan nanosphere matrix. Moreover, the decline in intensity and convergence of PAMTP-specific peaks in the range of 1250–1030 cm^−1^ demonstrated that PAMTP was caught and uniformly scattered at the molecular scale within the chitosan network. Its amino and hydroxyl groups bind to both therapeutic agents, PAMTP and Tyr, using hydrogen bonding, ionic interactions, and dipole–dipole attractions to form a biocompatible delivery vehicle. The FTIR evidence that supports this redirection of nanoparticles within their HDC matrix also supports the successful encapsulation of both therapeutic agents, the molecular interaction between them, and their hybrid payload within the chitosan nanoparticle network.

Dynamic light scattering (DLS) and zeta potential analyses of En@PAMTP_Tyr provided a means of assessing the physicochemical properties of the nanocarrier. The particle size distribution shown in Figure 2B indicates a narrow monomodal distribution with an average hydrodynamic diameter of 140.55 ± 21.26 nm and a polydispersity index (PDI) of 0.23 ± 0.04. The PDI value below 0.3 confirms that the nanocarrier particles exhibit a relatively uniform and homogeneous size distribution, indicating successful formation of monodisperse nanoparticles suitable for pharmaceutical applications. This indicated that nanocarrier particles with a uniform size distribution were successfully formed. Notably, the present study had a limitation in using DLS measurements only on freeze-dried En @PAMTPTyr re-dispersed nanoformulation and it could not quantitatively demonstrate any alteration in particle size or polydispersity index due to the freeze-drying step. Notably, the present study had a limitation in using DLS measurements only on freeze-dried En@PAMTP_Tyr re-dispersed nanoformulation and it could not quantitatively demonstrate any alteration in particle size or polydispersity index due to the freeze-drying step.

Zeta potential analysis (Figure 2C) indicated that the new nanoformulation had a surface charge of approximately +26.95 ± 1.23 mV. This charge provides good colloidal stability, mainly because they have a surface charge that causes them to repel one another. These positive charges arise mainly from the protonated amino groups on the HDCS surface. HDCS also provide good mucoadhesion and cellular uptake properties. Furthermore, a high surface charge is advantageous for interacting with negatively charged biological membranes. In summary, the dimensions and surface charges indicated that this nanoformulation (En@PAMTP_Tyr) had excellent physicochemical properties for penetrating tissues and delivering drugs to the target site.

### 3.3. Morphological Characterization

Surface morphology and shape of En@PAMTP_Tyr nanoformulation were examined by scanning electron microscopy (SEM) and transmission electron microscopy (TEM) (Figure 3). The SEM image of the freeze-dried powder (Figure 3A) showed nanoparticles embedded in a porous matrix and some discrete particles, which were observed as mostly quasi-spherical with only a slight agglomeration behaviour, which was common for chitosan–TPP systems after lyophilisation. The particles had smooth to texture surface, which is consistent with continuous polymeric shell composed of TPP-crosslinked highly deacetylated chitosan network, and their apparent dimensions qualitatively confirmed with our sub-200 nm size obtained by DLS [51]. Noteworthy, the slight irregularities observed in SEM for the quasi-spherical shape may be attributed to the structural modifications resulting from the freeze-drying process and the conventional ionotropic gelation method employed, wherein the TPP solution was added dropwise to the chitosan-drug dispersion rather than reverse addition, which can impact the uniformity of the particle shape upon drying.

High-resolution TEM further validated the nanoscaled size and shape of the En@PAMTP_Tyr, offering a more detailed view of its internal structure. As revealed by TEM nanograph (Figure 3B), the prepared particles appeared well-defined, predominantly spherical in shape with a uniform size less than 200 nm and each particle possessed a dark electron-density core surrounded by correspondingly lighter shell, possibly suggesting the encapsulation of PAMTP and Tyr in the chitosan matrix. Absence of large aggregates or irregular fragments in the TEM images substantiates the conclusion that both ionotropic gelation processes yield structurally homogenous nanocarriers, and that freeze-drying/redispersion procedure preserved their integrity to a degree compatible with an effective tissue penetration and brain delivery [51].

The EDX mapping images (Figure S7, ESM) provided insights into the elemental composition of the nanoparticles on a spatial basis. The presence of carbon (C), nitrogen (N), and oxygen (O) across the surface of the nanoformulation was confirmed by the uniform distribution of these elements, and their existence also indicated that they were part of the structural framework of the chitosan biopolymer. A consistent and clear signal from sulfur (S) across the surface of the nanoformulation can suggest the PAMTP encapsulation. The relatively low P and Na signals indicate the removal of excess unreacted TPP during the washing procedure and the incorporation of TPP as an ionic crosslinker in the nanoparticle structure. The even distribution of all mapped elements, especially S and P, which were found only in PAMTP and TPP, respectively, strongly supports our hypothesis that both therapeutic agents were homogeneously encapsulated and structurally integrated within the core of our nanoparticles. Overall, the SEM and EDX data confirmed that the En@PAMTP_Tyr nanoformulation was morphologically uniform, chemically well-defined, and compositionally homogeneous.

### 3.4. Pharmacological Characterization

The performance of dual-drug-loaded chitosan nanoparticles was evaluated using two parameters: drug entrapment efficiency (*EE*%) and loading capacity (*LC*%). Results show high drug encapsulation. *EE* percentages were PAMTP 73.45 ± 1.50% and L-tyrosine 90.85 ± 2.15%, indicating a strong affinity for the chitosan matrix, especially L-tyrosine, due to enhanced hydrogen bonding and ionic interactions with protonated amine groups of highly deacetylated chitosan. *LC*% for PAMTP and Tyr was 19.25 ± 1.30% and 28.95 ± 1.75%, respectively, showing the chitosan-based nanocarrier system’s high drug loading capacity. These results illustrate that efficient ionomeric gelation permits encapsulation of both hydrophilic and lipophilic molecules into the nanoparticle core. High loading and encapsulation values confirmed structural compatibility between drug molecules and the chitosan polymer. Importantly, these results support the system’s ability to co-deliver two neuroprotective agents at therapeutic doses. Thus, the platform shows promise for actual neurodegenerative disease treatment.

The in vitro release of Tyr and PAMTP from En@PAMTP_Tyr Nanoformulation was studied under two physiological conditions in PBS at pH 5.4 and pH 7.4, simulating acidic and neutral environments. Tyr exhibited a two-phase kinetic profile, as shown in Figure 4A. At pH 5.4, an initial burst release of 35% in the first 6 h was followed by a sustained release, reaching 78%, slightly higher with the polysaccharide carrier than with the control. Figure 4A shows that pH-modulated release enhanced drug diffusion from the swollen chitosan matrix. At pH 7.4, Tyr release was slower, with only 31% released after 48 h. Figure 4B shows PAMTP release kinetics, with a maximum cumulative release of ~90% at pH 5.4 by 48 h, but not exceeding 70% at pH 7.4. These results indicate pH-sensitive matrix behavior due to protonation of amino groups in the deacetylated chitosan backbone at lower pH, causing electrostatic repulsion, matrix swelling, and increased porosity, enhancing encapsulated molecule diffusion.

En@PAMTP_Tyr exhibited significant pH-responsive behaviour, releasing more PAMTP and L-tyrosine at acidic pH (5.4) than at physiological pH (7.4). Similar findings occur with chitosan-based nanocarriers, which release drugs in endosomal/lysosomal compartments and inflamed tissue via protonation and matrix swelling at mildly acidic pH [52], reducing premature leakage in systemic circulation. Crosslinking with TPP is ionic and pH dependent. At physiological pH (7.4), the chitosan–TPP complex is quite stable due to the strong electrostatic interaction between deprotonated TPP and partially protonated chitosan amino groups, and thus a compact matrix structure is formed to restrict drug diffusion. In contrast, at acidic pH (5.4), the protonation of chitosan amino groups is increased leading to a stronger electrostatic repulsion between adjacent polymer chains which results in swelling of the matrix, higher porosity and improved drug release. This pH-responsive release might explain the reported antidepressant-like, antioxidant, anti-inflammatory, and neuroprotective effects [53] in hippocampal tissue by enhancing local availability of PAMTP and Tyr in regions of oxidative stress and neuroinflammation, while gradual release at pH 7.4 ensures systemic tolerability. However, we do not measure pH gradients or intratissue release in vivo, making the connection between acid-boosted release and outcomes inferential, requiring pharmacokinetic and biodistribution studies.

The released pH-responsive profile is consistent with the literature on chitosan–TPP nanoparticle systems. For example, research has shown that chitosan–TPP nanoparticles release drug much more rapidly at acidic pH than at physiological pH because protonation destabilises the matrix. For example, Nejabat et al. [54] reported that the release of insulin from chitosan–TPP nanoparticles was 92% at pH 4.0 and 56% at pH 7.4 after 48 h, which was attributed to the increased chain opening and electrostatic repulsion at acidic conditions. Similarly, Wu et al. [55] demonstrated the pH sensitivity of resveratrol-loaded CS-TPP nanoparticles, which showed a better cumulative drug release at pH 6.5 (tumour tissue simulation) than at pH 7.4 (physiological condition).

This pH-dependent behaviour is due to protonation of amino groups of chitosan (pKa ≈ 6.5) at acidic pH which increases the electrostatic repulsion between the polymer chains and reduces the ionic crosslinking with TPP, leading to matrix swelling and increased drug diffusion. This feature is particularly beneficial for targeted delivery to pathological sites with acidic microenvironments, such as inflamed tissues (pH 5.5–6.5) and endosomal/lysosomal spaces (pH 4.5–6.0), where faster release can boost local therapeutic effect without premature systemic exposure [56,57,58]. Eventually, our release profiles (78% for Tyr and 90% for PAMTP at pH 5.4 vs. 31% and 70%, respectively, at pH 7.4 after 48 h) are in good agreement with these reported trends and confirm the successful development of a pH responsive nanocarrier appropriate for targeted delivery to acidic microenvironments associated with neuroinflammation and oxidative stress in depression pathology.”. This revision now clearly contrasts the behavior at pH 7.4 (physiological) versus pH 5.4 (acidic) and explains the mechanism at both conditions.

### 3.5. Behavioural and Biological Assessment

Behavioral assessments confirmed unpredictable stress-like depression induction, with sucrose preference and novel object discrimination indicating stress-induced anhedonia, improved by pre-treatment with chitosan nanoparticles encapsulating En@PAMTP_Tyr. Free PAMTP and L-tyrosine were not tested because L-tyrosine is metabolized by enzymes, such as tyrosine aminotransferase, reducing its brain bioavailability. Free L-tyrosine showed limited antidepressant effects in rats, whereas L-tyrosine-loaded nanoparticles enhanced catecholamine restoration in behavioral tests [21,59]. Similarly, PAMTP, a lipophilic MAO-B inhibitor analogue, benefits from nano-encapsulation to improve metabolism and BBB penetration. Our co-encapsulated En@PAMTP_Tyr composite was chosen for synergistic effects, shown by its pH-responsive release and outcomes comparable to selegiline [60]. Selegiline, mainly oral in clinical use, has low bioavailability (4–10%) due to first-pass metabolism, limiting brain exposure and increasing side effects. The new chitosan nanoparticles protect the drug, allow pH-responsive release, and enhance blood–brain barrier penetration through a positive charge and tight junction modulation. These features make En@PAMTP_Tyr a promising platform for better CNS targeting with less peripheral exposure than oral MAO inhibitors [61,62].

#### 3.5.1. Sucrose Preference, Body Weight, Tail Suspension, Discrimination Index (DI), Passive Avoidance, and Grip Strength

The CUSD_En@PAMTP_Tyr group showed higher sucrose preference than the CUSD group (*p* < 0.0001), indicating a reversal of stress-induced anhedonia. The selegiline group also had higher sucrose preference than CUSD (*p* < 0.0001) and similar levels to En@PAMTP_Tyr and CUSD_En@PAMTP_Tyr, suggesting the composite’s efficacy (Figure 5A). Differences across groups were due to lower sucrose preference in CUSD, confirming treatment effectiveness against chronic unpredictable stress depression (CUSD) reduction. Body weight was measured, with CUSD showing a significant decrease compared to the negative control (*p* < 0.0001), confirming stress-induced weight loss. CUSD_En@PAMTP_Tyr had higher body weight than CUSD (*p* < 0.001), and selegiline showed no significant difference from the negative control or CUSD. CUSD_En@PAMTP_Tyr did not significantly alter body weight compared to selegiline, indicating that these treatments maintained normal weight (Figure 5B).

The present study examined rat immobility time during the tail suspension test across five groups for CUSD-like behavior and treatment effects. The negative control group had the lowest immobility time, whereas the CUSD group showed a significant increase, confirming depression induction (*p* < 0.0001). CUSD_EN_PAMTP_Tyr administration significantly reduced immobility time compared to the CUSD group (*p* < 0.0001). No significant differences were found between CUSD_EN_PAMTP_Tyr and selegiline treatments, suggesting different mechanisms or efficacy levels (Figure 5C). The study also evaluated En@PAMTP_Tyr on cognitive memory and neuromuscular function in a CUSD-like model. In the novel object discrimination task (Figure 5D), CUSD animals showed a significant decrease in the discrimination index compared to controls (*p* < 0.0001), indicating cognitive impairments. En@PAMTP_Tyr administration significantly improved object discrimination in the CUSD_En@PAMTP_Tyr group compared to the untreated CUSD group (*p* < 0.0001). This improvement suggests enhanced cognitive abilities and memory function, which are important for cognitive decline research [63]. En@PAMTP_Tyr significantly boosted grip strength in the CUSD group with *p* < 0.01, while Selegiline with *p* < 0.05, but was less effective than En@PAMTP_Tyr (Figure 5E). The efficacy of En@PAMTP_Tyr is due to its active component and nanoparticle delivery system, which improves neurotransmission in mood, cognition, and reward regions [13]. Tyrosine may enhance dopamine and norepinephrine production, thereby improving mood and cognitive function [64].

Depression is associated with neuromuscular impairment, such as psychomotor retardation, decreased muscular endurance, and reduced grip strength, indicating central and peripheral dysregulation. Dopaminergic pathways to the striatum and motor cortex modulate voluntary motor output. Depletion of striatal dopamine, observed with elevated MAO-B activity in CUSD-exposed animals, impairs motor behaviour, contributing to diminished grip force in untreated depression-model rats. Restoring dopaminergic signaling through MAO-B inhibition by PAMTP is expected to partially ameliorate this central motor deficit [65]. Furthermore, elevated AChE activity in the CUSD group suggests impaired neuromuscular junction efficiency and reduced force generation and its normalizing activity with En@PAMTP_Tyr treatment, supported by biochemical data and docking affinity, explains the recovery in grip strength [66].

Jongkees et al. [64] showed that encapsulated L-tyrosine enhances adrenergic pathways linked to autonomic skeletal muscle tone and stress-related motor performance, supporting En@PAMTP_Tyr’s role in boosting grip strength in treated animals.

#### 3.5.2. Aversive Learning and Memory Retention (Passive Avoidance Test)

The passive avoidance task assesses memory by evaluating an animal’s ability to avoid an area where it encountered an unpleasant stimulus. During training (Figure 6A), no notable differences were observed in the time to enter the dark chamber among groups, except for the healthy group pre-treated with En@PAMTP_Tyr, which differed significantly (*p* < 0.05) from the CUSD group. However, 30 min later, CUSD rats entered the dark compartment faster than the control group (*p* < 0.05), indicating impaired memory. Both healthy rats pre-treated with En@PAMTP_Tyr and CUSD_En@PAMTP_Tyr showed longer latencies than the CUSD group (*p* < 0.01). The performance of the CUSD_En@PAMTP_Tyr group was comparable to that of the selegiline group, with similar improvements over the CUSD group (*p* < 0.0001; Figure 6B).

Therefore, chitosan nanocarrier delivery of En@PAMTP_Tyr revealed memory enhancement to aversive stimuli in depressed animals [6].

In the acquisition trial, the healthy pre-treated group took longer to enter the dark compartment than the CUSD group, indicating enhanced aversive learning acquisition with En@PAMTP_Tyr without prior stress. This suggests that MAO-B inhibition and catecholaminergic support in neurologically intact animals improve aversive experience encoding, possibly through dopamine-dependent modulation of fear memory circuits in the hippocampus and amygdala [67]. Similarly, selegiline in non-depressed models showed increased dopaminergic activity enhanced attention and associative learning. This acquisition effect did not impair avoidance or cause hyperactivity; the healthy pre-treated group showed strong retention at 30 min, indicating normal memory consolidation [13].

### 3.6. Antioxidant Activity Assays

The antioxidant properties of the En@PAMTP_Tyr nanocomposite were assessed by examining the effect of superoxide dismutase (SOD) in neutralizes free radicals. Administration of En_PAMTP_Tyr to healthy animals resulted in upregulation of SOD levels in hippocampal homogenates with significance higher than those in the chronic unpredictable stress depression (CUSD) group (*p* < 0.001) and slightly higher than those in the control group, indicating antioxidant properties. The CUSD_ENPAMTP_ Tyr-treated group showed upregulation of SOD compared to both the CUSD (*p* < 0.001) and selegiline-treated groups (*p* < 0.01), underscoring the superior antioxidant capacity of the composite (Figure 7A). Additionally, Figure 7B displays hippocampal glutathione (GSH) levels in the experimental groups. The CUSD group showed a significant GSH decrease compared to controls, underscoring the oxidative stress–depression link. CUSD_En@PAMTP_Tyr animals had elevated GSH levels, similar to control group. The lack of significant difference in GSH levels between CUSD and CUSD + En@PAMTP_Tyr does not imply treatment inefficacy; as, PAMTP a 4-methylthiophenyl moiety that is not a direct free radical scavenger or GSH-regenerating agent. Its thioether group offers modest redox modulation. The formulation has a main antioxidant effect, which significantly impacts superoxide dismutase (SOD) activity and reduces MAO-mediated hydrogen peroxide production [68]. This indicates the protective or restorative role of the compound in oxidative stress. Animals receiving selegiline showed no notable increase in GSH levels compared to the depressed group, suggesting that depression significantly reduces brain GSH levels. Although En@PAMTP_Tyr treatment aids in the restoration of antioxidant capacity in depression-induced oxidative stress, it lacks an antioxidant structure–activity linked to phenols and catechols. The 4-methylthiophenyl group may slightly modify the molecule’s redox properties but is not a strong ROS-scavenging pharmacophore. It features a thiol group characterized by sulfhydryl (−SH) groups that enhance antioxidant properties through ROS scavenging, cellular redox homeostasis, and oxidative damage protection (Ulrich & Jakob, 2019) [69].

The study assessed AChE activity in depression-induced groups to explore cholinergic signalling in cognitive control. In CUSD animals, AChE activity was significantly higher than that in controls (−ve C; *p* < 0.0001), indicating that excessive acetylcholine breakdown may be related to depression-related deficits (Figure 7C). Depressed animals treated with En@PAMTP_Tyr (CUSD_En_PAMTP_Tyr) showed a significant reduction in AChE activity compared to untreated CUSD (*p* < 0.0001), suggesting the restoration of normal cholinergic function. AChE levels in the CUSD_En@PAMTP_Tyr group matched those in the controls, with no significant change. While selegiline slightly reduced AChE activity in depression, CUSD_En@PAMTP_Tyr more effectively normalized AChE activity. These findings suggest that encapsulated nanochitosan PAMTP_Tyr may modulate mood-affecting neural circuits, making it a promising candidate for depression-related cholinergic dysfunction [70].

The activity of monoamine oxidase (MAO) was assessed as a biomarker of oxidative stress, crucial in depression development. The depression-induced group (CUSD) showed a significant increase in MAO activity (*p* < 0.0001) compared to the negative control, indicating depressive states may enhance oxidative metabolism and MAO expression. Notably, both the CUSD_En@PAMTP_Tyr and selegiline groups had significantly reduced MAO levels (*p* < 0.0001) compared with CUSD rats, showing that En@PAMTP_Tyr mitigated the MAO elevation linked to depression (Figure 7D). These findings show that depression increases MAO activity, contributing to oxidative stress and neurotransmitter imbalance.

Inhibition of MAO-B offers neuroprotection by reducing oxidative stress and inflammation, damaging neurons in depression, and restoring synaptic plasticity for aversive memory [6,13]. The En@PAMTP_Tyr nanocarrier showed improved bioavailability and prolonged latency in avoiding aversive conditions, indicating its potential for treating depression-related cognitive impairments and functioning as a selective, irreversible MAO-B inhibitor due to its propargylamine group, which permanently bonds with the enzyme’s active site [71]. This group irreversibly binds to the FAD cofactor in MAO-B’s catalytic domain, inhibiting dopamine breakdown and reducing oxidative byproducts like hydrogen peroxide. The dual mechanism of preserving dopamine and reducing oxidative stress shows the compound’s therapeutic potential. The analog 2-N-propargylamino-1-(4-methylthiophenyl) propane has a 4-methylthio (-SCH_3_) moiety on the aromatic ring, affecting electronic density and lipophilicity compared to selegiline’s phenyl system, with a 2-(methyl(propargyl) amino) ethyl substituent on an unsubstituted phenyl ring [72].

Depression is associated with reduced brain antioxidants, such as glutathione (GSH) and superoxide dismutase (SOD). Oxidative imbalance is a hallmark of depressive disorders, as stress-induced reactive oxygen species (ROS) harm synaptic integrity [73]. Selegiline and En@PAMTP_Tyr increased GSH levels and inhibited monoamine oxidase (MAO) by lowering oxidative stress, potentially increasing enzyme activity and affecting neurotransmitter levels [39]. Antioxidant treatment can manage oxidative stress-induced upregulation of monoamine oxidase A (MAO-A) and B (MAO-B) [74]. En@PAMTP_Tyr upregulated monoamine oxidase inhibition, enhancing cognitive function by increasing neurotransmitter availability and providing neuroprotective effects through the thiolphenyl group, which enhances blood–brain barrier penetration and binding to enzymes, receptors, and transporters [75]. Research on monoamine oxidase inhibition holds potential for improved cognitive impairment treatments. Kim and Kim [76] showed that grip strength and physical performance in depressed rats improved with selegiline, but En@PAMTP_Tyr had superior outcomes. En@PAMTP_Tyr treatment resulted in prolonged latency, indicating improved memory retention. The post-depression treatment group surpassed the pre-treatment group, showing greater benefits of En@PAMTP_Tyr during depression-stress.

### 3.7. Molecular Docking Simulation

The bioactivity of 2-N-Propargylamino-1-(4-methylthiophenyl) propane (PAMTP) was evaluated against monoamine oxidase and acetylcholinesterase receptors and compared with selegiline under identical molecular docking parameters. Both PAMTP and selegiline demonstrated superior binding affinities for monoamine oxidase (ID: 3PO7) than for acetylcholinesterase (ID: 5FPQ). As illustrated in Table 1, PAMTP exhibited a free energy of binding of −6.23 Kcal/mol and an inhibition constant (Ki) of 26.98 µM with monoamine oxidase, while with acetylcholinesterase, it recorded −3.75 Kcal/mol and a Ki of 1.8 mM. Conversely, selegiline showed a free energy of binding of −5.96 Kcal/mol and a Ki of 42.75 µM with the monoamine oxidase receptor and −3.54 Kcal/mol and a Ki of 2.55 mM with acetylcholinesterase. The computational results suggested that the bioactivities of PAMTP and selegiline were comparable. However, PAMTP demonstrated slightly superior complexation with both receptors in terms of the free binding energies and inhibition constants. As depicted in Figure 8 and Figure 9, the resulting noncovalent interactions (NCIs) include hydrogen bonding, van der Waals forces, and electrostatic attraction. As detailed in Table 1, PAMTP forms one hydrogen bond during its complexation with both monoamine oxidase and acetylcholinesterase through TYR435 and ASP306 amino acids, respectively. Selegiline forms a single hydrogen bond during its complexation with monoamine oxidase through the GLY434 amino acid.

In silico analysis shows a hydrogen bond with TYR435, supporting PAMTP’s orientation, consistent with propargylamine–MAO-B complexes [6]. Biochemical data confirm this. CUSD-exposed animals had higher hippocampal MAO activity than controls (*p* < 0.0001), indicating stress-induced MAO-B upregulation, monoamine catabolism, and hydrogen peroxide generation. En@PAMTP_Tyr treatment significantly reduced MAO activity (*p* < 0.0001), akin to selegiline, suggesting a compound with greater MAO-B affinity, delivered via a cationic chitosan nanocarrier. The convergence of in silico binding affinity and in vivo inhibition validates two evidence levels [12]. Restoring dopaminergic activity through MAO-B inhibition correlates to an antidepressant phenotype affecting reward processing and motivation. The CUSD + En@PAMTP_Tyr group showed significant sucrose preference improvement versus untreated CUSD animals (*p* < 0.0001) due to MAO-B inhibition, reducing dopamine breakdown and preserving synaptic tone [74].

Reduced tail suspension immobility time aligns with pro-dopaminergic and pro-serotonergic effects of MAO-B inhibition, linked to stress-coping behaviors. Conversely, PAMTP’s weaker AChE binding suggests partial inhibition [37]. This site affects enzyme kinetics, targeted by dual-binding site inhibitors in neurodegenerative disease therapeutics [11]. Biochemical evidence supports this. AChE activity in hippocampal tissue under CUSD was higher than that in controls (*p* < 0.0001), consistent with cholinergic enzyme hyperactivation in chronic stress and depression-related neurodegeneration [10]. Increased AChE activity accelerates acetylcholine hydrolysis, impairing neurotransmission, contributing to cognitive deficits in depression and Alzheimer-like pathology [47]. En@PAMTP_Tyr restored AChE activity to control levels (*p* < 0.0001 vs. CUSD), surpassing selegiline’s efficacy. Although potent AChE inhibition cannot be inferred from weak docking affinity, hydrogen bonding with ASP306 and biochemical normalization suggest PAMTP modulates cholinergic enzyme function via nanoparticle delivery. The cognitive significance of AChE normalization by En@PAMTP_Tyr is evident in memory-sensitive tasks, with a higher novel object discrimination index than untreated CUSD animals, indicating restored hippocampus-dependent memory [63]. En@PAMTP_Tyr’s normalization of AChE activity preserves synaptic acetylcholine, facilitating these processes. In the passive avoidance test, CUSD + En@PAMTP_Tyr-treated animals showed significantly extended latencies to enter the dark compartment compared to untreated CUSD animals (*p* < 0.01), aligning with restored cholinergic function in hippocampal memory circuits [77].

### 3.8. Anti-Inflammatory Activity of En@PAMTP_Tyr for TNF-α and IL-10 Cytokines

TNF-α and IL-10 levels were evaluated using ELISA to assess cytokines in relation to depression and the effects of En@PAMTP_Tyr as an immunomodulatory treatment. The depression group (CUSD) had the lowest IL-10 levels, a crucial anti-inflammatory cytokine, which were significantly lower than those in the negative control (*p* < 0.0001). The CUSD_En@PAMTP_Tyr group showed increased IL-10 expression (*p* < 0.0001) compared to the untreated depression group. The selegiline group had moderately elevated IL-10 levels compared to the depression group (*p* < 0.01) (Figure 10A). Moreover, Figure 10B shows that the depression group (CUSD) had higher TNF-α levels than the negative control (*p* < 0.01). Depression-treated animals (CUSD_En@PAMTP_Tyr) showed improvement, with a notable reduction in TNF-α, which was significantly lower than that in the untreated group (*p* < 0.001). The selegiline-treated group had reduced TNF-α levels compared to those in depression-induced rats (*p* < 0.05).

Additionally, the link between depression and inflammation, highlighting elevated TNF-α levels in behavioral and neurochemical modifications [78]. Our findings showed that animals with depressive symptoms had increased TNF-α and decreased anti-inflammatory IL-10 levels, likely as a compensatory response. En@PAMTP_Tyr administration significantly reduced TNF-α to nearly baseline in the negative control group [79]. Concurrently, IL-10 levels normalized, indicating reduced inflammation and restored immune balance, which is crucial for inflammation-related depression [78]. Selegiline also reduced cytokine levels; however, the effects were less pronounced (*p* < 0.05) than those in the negative control group.

### 3.9. Histological Analysis of Apoptotic and Degenerate Cells in the CA1 Region of Rat Hippocampus

In models of depression, such as those induced by chronic unpredictable stress depression (CUSD), the CA1 region of the rat hippocampus is analyzed using hematoxylin and eosin (H&E) staining. As illustrated in Figure 11A, hippocampal sections from the control group showed normal, tightly packed pyramidal neurons with distinct nuclei. In contrast, the depression-induced group (CUSD) exhibited neuronal degeneration, shrinkage, and the formation of pyknotic nuclei. Apoptotic and degenerated cells with nuclear condensation, a hallmark of apoptosis, and cytoplasmic shrinkage and vacuolization indicated degeneration. In the CA1 pyramidal cell layer, apoptotic cells had intensely stained shrunken nuclei with chromatin condensation and pyknotic nuclei with fragmented chromatin and pale, eosinophilic cytoplasm (Figure 11B).

The En@PAMTP_Tyr group (CUSD_ En@PAMTP_Tyr) maintained a neuronal structure similar to that of the control group. The study found that animals showing signs of depression benefited from notable neuroprotective effects after receiving En@PAMTP_Tyr treatment, with protective action on the structure of pyramidal neurons from all induced stresses. In contrast, the group that received selegiline experienced moderate neuronal recovery with less cellular disorganization. These results indicate that encapsulated PAMTP-Tyr effectively protects neuronal integrity under depressive stress conditions.

### 3.10. Immunohistological Analysis of Tau Protein, β-Amyloid Aggregations, and BDNF in the CA1 Region of Rat Hippocampus

Figure 12I illustrates an uneven distribution of immunoreactions, characterized by varying intensities of tau protein, β amyloid aggregations (Figure 12II), and brain-derived neurotrophic factor (Figure 12III), all of which are concentrated in fibrous or matrix-like structures. Tau, a microtubule-associated protein, is essential for maintaining cytoskeletal integrity and facilitating axonal transport. Its hyperphosphorylation leads to oxidative modifications linked to neurodegenerative and depressive disorders, which subsequently cause aggregation.

Figure 12I(B) shows reduced tau expression in the depression group, indicating neuronal destabilization. In contrast, the control group exhibited the highest tau expression, which is crucial for normal neuronal functions. The depression group displayed a significant reduction in tau levels (*p* < 0.0001), suggesting microtubule instability. Conversely, the CUSD_En@PAMTP_Tyr and selegiline groups showed significant tau upregulation compared to the CUSD group (*p* < 0.0001) (Figure 12IV(A)). En@PAMTP_Tyr may modulate tau phosphorylation, prevent oxidative damage, maintain tau integrity, reduce aggregation, and decrease ROS-induced neurotoxicity. However, Wang et al. [80] noted that tau has a complex relationship with depression and is not directly involved in all forms of the condition, particularly late-life depression. Understanding tau upregulation is critical for developing therapies for neurodegenerative diseases and mood disorders, and this research highlights the necessity of exploring tau’s role in brain health. Figure 12II(B) reveals elevated-amyloid aggregation in the depression group, which was significantly reduced by En@PAMTP_Tyr. This suggests that the treatment interferes with β-amyloid generation or aggregation, possibly by modulating APP processing or enhancing autophagic clearance. Furthermore, the antioxidant potential of En@PAMTP_Tyr may counteract lipid peroxidation and the oxidative stress-induced upregulation of β-secretase, a central factor in β-amyloid production [81]. The reduction in β-amyloid burden was more pronounced with En@PAMTP_Tyr than with selegiline, suggesting a more extensive regulatory effect on cellular redox balance and proteostasis through enhanced glutathione and SOD activity. Finally, Figure 12IV(B) shows significantly higher amyloid levels in the depression group compared to the control group (*p* < 0.0001), while the CUSD_En@PAMTP_Tyr group showed a significant decrease in aggregate expression (*p* < 0.0001).

**Figure 12:**
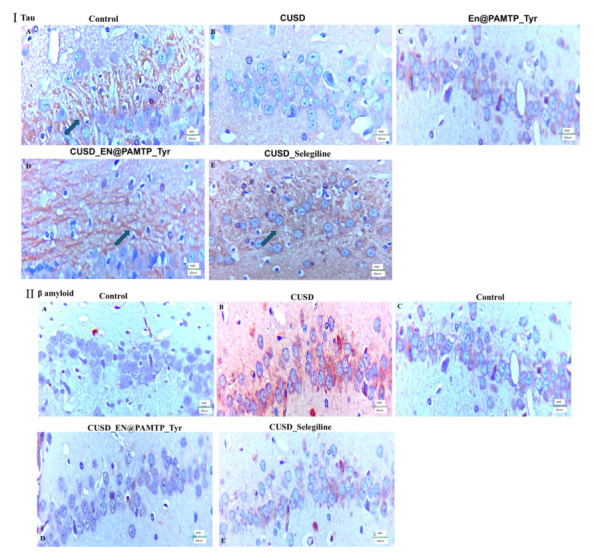

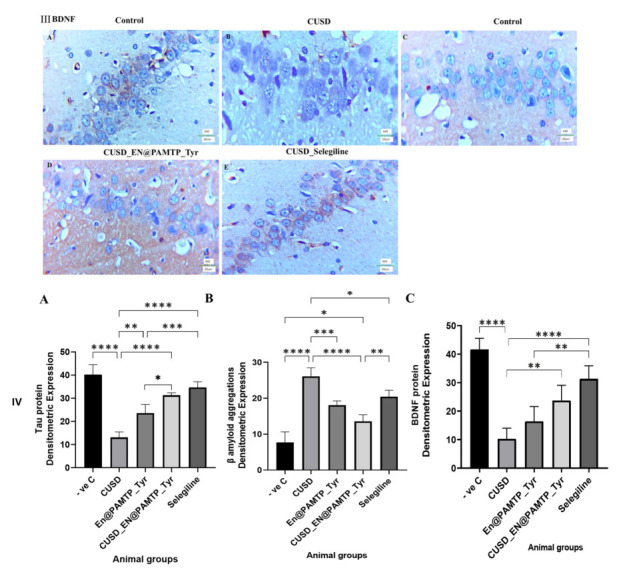
Immunohistochemical analysis of tau, β-amyloid, and BDNF expression in hippocampal sections across the experimental groups. (**I**) Representative micrographs (scale bar = 20 μm) show the expression levels of tau (**I**), β-amyloid (**II**), and BDNF (**III**) in the control, depression, En@PAMTP_Tyr, CUSD_En@PAMTP_Tyr, and selegiline-treated groups. (**IV**) Quantitative densitometric analysis of hippocampal tau, β-amyloid, and BDNF protein expression across the experimental groups. (**A**) Tau protein expression, (**B**) β-amyloid aggregation levels, and (**C**) BDNF protein expression levels were measured and compared. Data are presented as the mean ± SEM. Statistical significance is indicated as * *p*  <  0.05, ** *p*  <  0.01, *** *p*  <  0.001, **** *p*  <  0.0001.

#### 3.10.1. Depression-Associated Neuropathology and Its Reversal by En@PAMTP_Tyr

The neurobiological effects of chronic depression go beyond monoaminergic dysregulation, including Alzheimer-like changes such as β-amyloid accumulation, disrupted tau protein expression, and reduced brain-derived neurotrophic factor (BDNF), which collectively harm hippocampal integrity and neuroplasticity [82].These changes result from sustained oxidative stress and impaired protein degradation pathways, which are the hallmarks of the chronic unpredictable stress depression (CUSD) model. CUSD-exposed animals showed significantly elevated β-amyloid aggregation, reduced tau expression, and suppressed BDNF levels compared to controls, confirming the neurodegenerative aspect of the model. En@PAMTP_Tyr treatment significantly reversed neuropathological changes. β-Amyloid aggregate levels in the CUSD_En@PAMTP_Tyr group were much lower than those in the untreated depression group, with a reduction greater than that achieved by free selegiline (*p* < 0.05), highlighting the superior efficacy of nanoparticle encapsulation and co-delivered tyrosine [80]. Tau protein expression was also normalized, restoring cytoskeletal integrity diminished by chronic stress. Although free selegiline reduced β-amyloid accumulation relative to the CUSD group (*p* < 0.001), its modest effect reflects limited CNS bioavailability and lack of complementary tyrosine. En@PAMTP_Tyr’s ability to normalize β-amyloid levels more effectively than an MAO-B inhibitor suggests a disease-modifying action, which is absent in most approved antidepressants [82].

The therapeutic activity of En@PAMTP_Tyr arises from its three components. PAMTP irreversibly inhibits MAO-B through its propargylamine moiety, reducing dopamine, serotonin, and noradrenaline catabolism, and sustaining monoaminergic tone in critical brain regions. Its 4-methylthiophenyl substituent and modified side chain may enhance CNS selectivity and enhances blood–brain barrier permeability and interacts with CNS targets, whereas the propargylamine group ensures irreversible inhibition [23]. These features define a CNS-directed pharmacophore relevant to antidepressant signaling [19]. L-tyrosine complements this by replenishing catecholamine pools depleted under stress [8]. It uses LAT1 at the blood–brain barrier for CNS uptake and sustained drug release, enhancing delivery while supporting catecholamine biosynthesis and cognitive performance [64,69]. This dual role underpins En@PAMTP_Tyr’s behavioral superiority over selegiline in memory tasks. The chitosan nanocarrier amplifies effects through antioxidant capacity, neuroprotection, and improved CNS bioavailability via electrostatic adhesion and transcytosis [83]. pH-responsive release at pH 5.5 targets pathologically active areas [84]. Encapsulation protects PAMTP and L-tyrosine from premature metabolism, extending half-lives, and maximizing CNS exposure [85]. These carrier-mediated effects likely underlie the efficacy observed here [86].

#### 3.10.2. Biphasic Preconditioning and Integrative Mechanistic Summary

An intriguing observation from the prophylactic administration of En@PAMTP_Tyr was a preconditioning phenotype with enhanced superoxide dismutase activity without reductive stress, suggesting a hormetic response where a low-level oxidative stimulus upregulates antioxidant defense systems [87]. This preconditioning maintained normal MAO activity, reduced β-amyloid aggregation, and preserved tau and BDNF expression, indicating the nanoformulation primes the brain’s redox defense before stress-induced changes [60]. Dual-phase antioxidant compounds that induce hormetic neuroprotection are valued in preclinical literature [87], suggesting En@PAMTP_Tyr warrants further investigation. These findings support a model where En@PAMTP_Tyr addresses depression-associated neuropathology. PAMTP inhibits MAO-B, preserving neurotransmission; L-tyrosine replenishes catecholamine precursors; chitosan-mediated antioxidant activity and CNS delivery reduce neuroinflammatory and oxidative burden; reduced oxidative stress restores MAPK/ERK, PI3K/Akt, and BDNF-TrkB signalling for neuroplasticity, synaptic maintenance, and cognitive function. This nanoparticle-enabled pharmacological profile across affective, cognitive, neuromuscular, and neuropathological dimensions distinguishes En@PAMTP_Tyr from conventional antidepressants and MAO inhibitors, providing a foundation for therapeutic improvements documented in this study [88].

## 4. Conclusions

This study highlights chitosan nanoparticle co-encapsulation of PAMTP and L-tyrosine as a multifaceted approach for treating depression beyond traditional monoamine enhancement. The formulation En@PAMTP_Tyr showed favorable nanoscale properties, dual-drug loading, and pH-responsive release, improving behavior in rats under chronic unpredictable stress (CUSD). En@PAMTP_Tyr enhanced resilience against anhedonia, despair-like behaviors, cognitive deficits, and motor impairments. It strengthened brain antioxidant defense, normalized monoamine oxidase (MAO) and acetylcholinesterase (AChE) activities, and restored cytokine balance (TNF-α and IL-10). It preserved hippocampal neuron integrity and reversed changes in tau, β-amyloid, and brain-derived neurotrophic factor (BDNF) levels linked to depression and neurodegeneration. En@PAMTP_Tyr’s primary advantage over traditional selegiline is its multifaceted nanotherapeutic design. Chitosan nanoparticles (~140 nm, +27 mV) facilitate co-delivery of PAMTP (a selegiline analogue) and L-tyrosine with high entrapment efficiency, pH-responsive sustained release, and improved blood–brain barrier penetration. This approach addresses selegiline’s limitations, like low oral bioavailability (~4%), peripheral metabolism, and restricted cognitive/neuroprotective effects, resulting in superior or comparable enhancements in anhedonia, cognition, oxidative stress, neuroinflammation, and hippocampal pathology in the CUS model. The comparable or superior efficacy to selegiline, coupled with molecular docking studies showing strong PAMTP interactions with MAO and AChE active sites, suggests its potential as a next-generation nanoformulation for major depressive disorder, particularly with cognitive impairments. Future research should focus on evaluating the long-term safety, pharmacokinetics, and applicability in other neurodegenerative models for clinical development.

## 5. Study Limitation

One of the main limitations of the study is the use of intraperitoneal administration, which is standard in rodent pharmacology but not viable for neuropsychiatric therapeutics. Future research should explore the efficacy of En@PAMTP_Tyr via oral or intranasal routes, the latter being promising for translation. Chitosan’s mucoadhesive and cationic properties suit nasal adhesion and olfactory neuron transport, enabling direct nose-to-brain delivery and bypassing the blood–brain barrier, aligning with clinical applications. Another limitation is the exclusive use of animal models, which means that the findings cannot be directly applied to humans, whose depression involves complex psychological and social factors. The study’s focus on a single compound under controlled conditions raises questions about long-term safety, dosing, side effects, and nanoparticle system performance in the complex human brain environment.

## Figures and Tables

**Figure 1:**
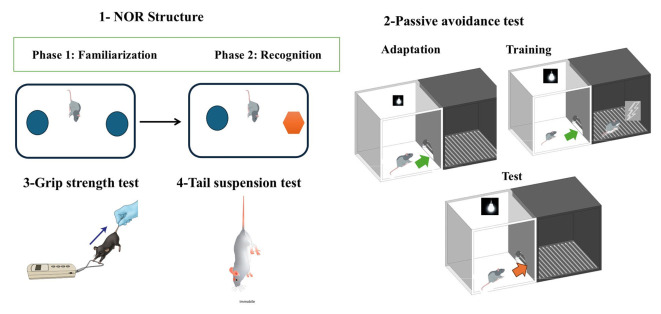
Schematic representation of the behavioral paradigms used in this study. 1—novel object recognition (NOR), 2—passive avoidance, 3—grip strength test, and 4—tail suspension test.

**Figure 2:**
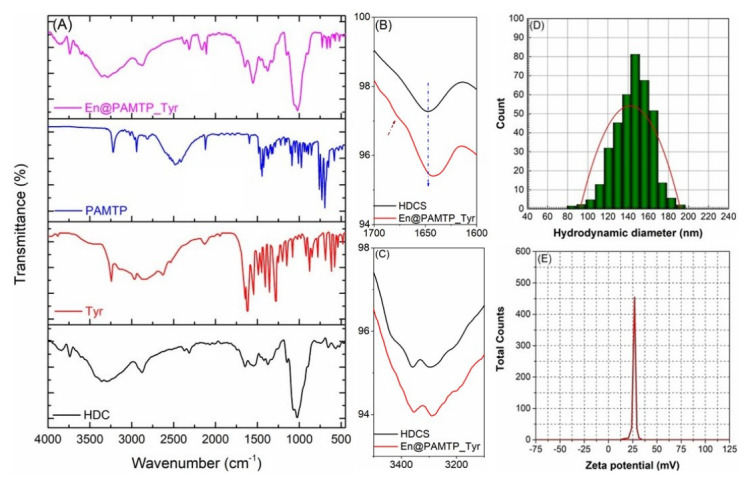
(**A**) FTIR spectra of HDCS, Tyr, PAMTP, and En@PAMTP_Tyr with expanded panel views showing: (**B**) the O-H/N-H stretching region (3500–3100 cm^−1^) and (**C**) the amide I region (1700–1600 cm^−1^). (**D**) Particle size distribution (PSD) of En@PAMTP_Tyr. (**E**) Zeta potential (ZP) of En@PAMTP_Tyr nanoformulation.

**Figure 3:**
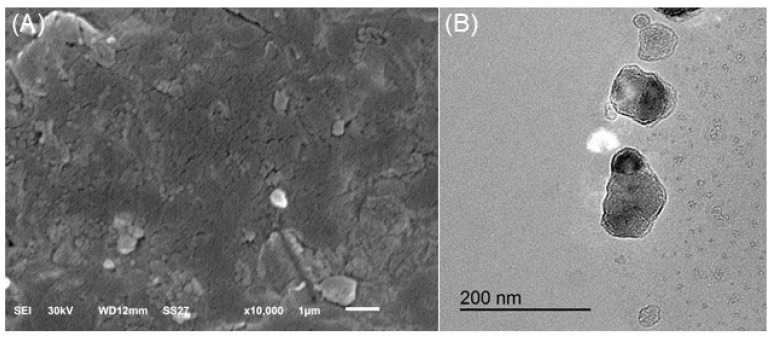
(**A**) SEM microimage and (**B**) TEM nanograph of En@PAMTP_Tyr nanoformulation.

**Figure 4:**
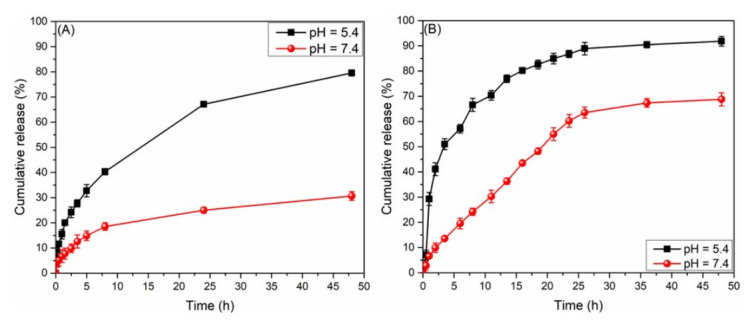
In vitro release profile of (**A**) Tyr and (**B**) PAMTP from their encapsulated form (En@PAMTP_Tyr) in buffer releasing media with different pH levels (5.5 and 7.4).

**Figure 5:**
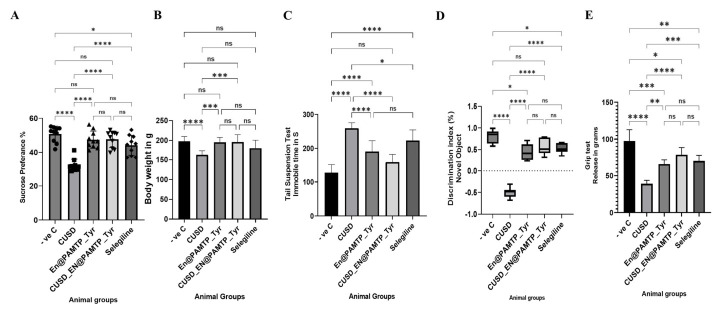
Evaluation of sucrose preference, body weight, cognitive performance, and neuromuscular function across the experimental groups. (**A**) sucrose preference %, (**B**) body weight change in grams, (**C**) tail suspension immobility time in seconds, (**D**) novel object discrimination index (%), (**E**) grip strength (release in g). Data are presented as the mean ± SEM. Statistical differences are indicated as * *p*  <  0.05, ** *p*  <  0.01,*** *p* < 0.001, and **** *p*  <  0.0001; ns = not significant.

**Figure 6:**
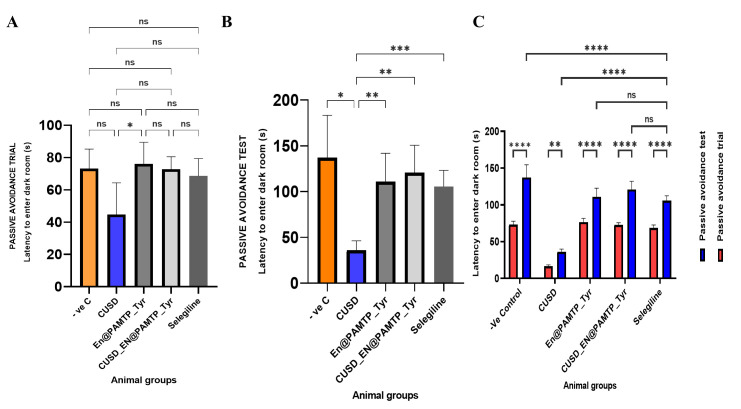
Behavioral assessment using the passive avoidance test in the experimental groups. The latency to enter the dark compartment is shown for (**A**) the acquisition trial, (**B**) the retention test, and (**C**) a comparative analysis between the two phases. Data are presented as the mean ± SEM. Statistical significance is denoted as * *p*  <  0.05, ** *p*  <  0.01,*** *p*  <  0.001, **** *p*  <  0.0001, ns, not significant.

**Figure 7:**
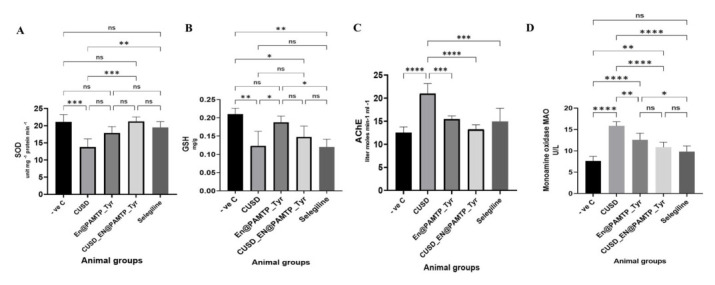
Effects of PAMTP_Tyr and Selegiline on SOD, GSH, AChE, and MAO activities in hippocampal tissue across experimental groups. Bar graphs represent mean ± SE values for (**A**) glutathione (GSH), (**B**) acetylcholinesterase (AChE), (**C**) superoxide dismutase (SOD), and (**D**) monoamine oxidase (MAO). Statistical significance indicated as * *p* < 0.05, ** *p* < 0.01, *** *p* < 0.001, **** *p* < 0.0001, ns, not significant.

**Figure 8:**
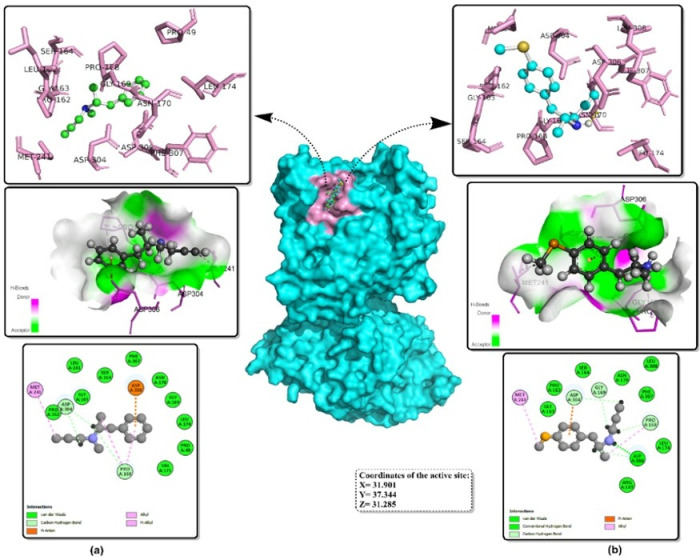
Binding pocket of (**a**) selegiline and (**b**) PAMPT inside the monoamine oxidase receptor (ID: 3PO7). The 3D and 2D visualizations, as well as the hydrogen bonding illustration through ligand–protein complexation of the best conformations, were obtained from the molecular docking simulations.

**Figure 9:**
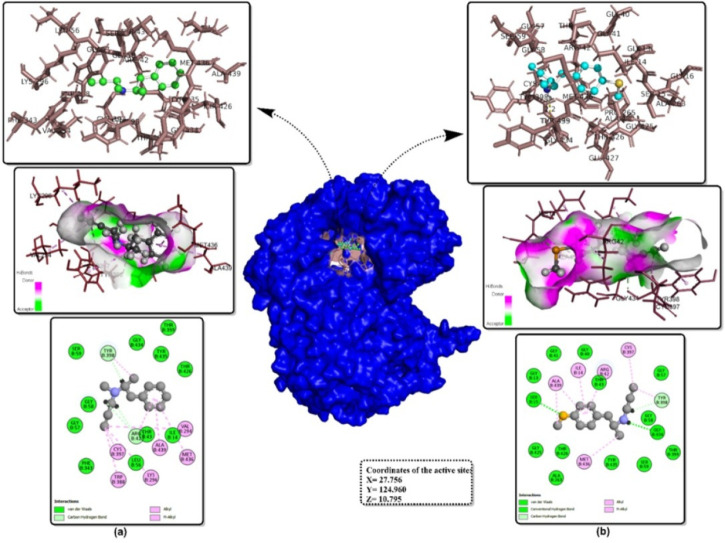
Binding pocket of (**a**) selegiline and (**b**) PAMPT inside the acetylcholinesterase receptor, ID: 5FPQ. The 3D and 2D visualizations, as well as the hydrogen bonding illustration through ligand–protein complexation of the best conformations, were obtained from the molecular docking simulations.

**Figure 10:**
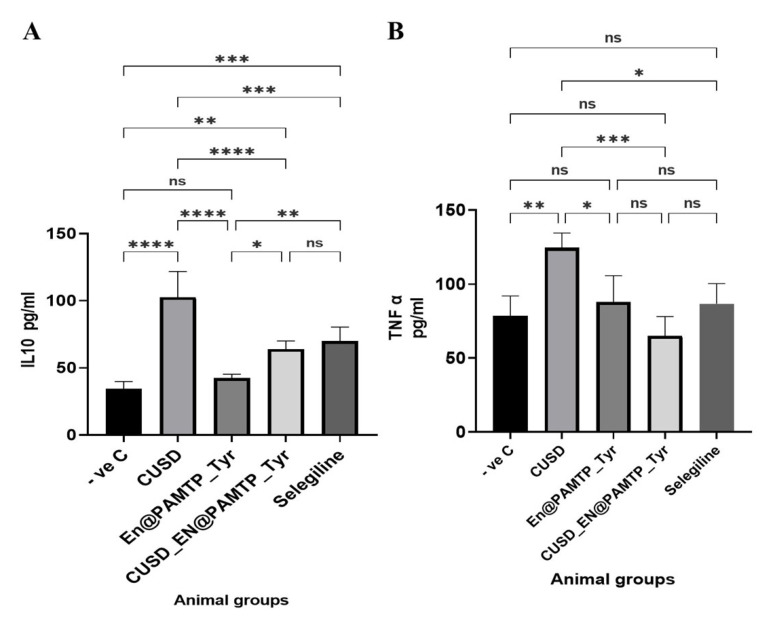
Quantitative expression of the inflammatory cytokines IL10 (**A**) and TNFα (**B**) after encapsulated PAMTP_tyrosine (En@PAMTP_Tyr) treatment in the CUSD model. Significance is expressed as the mean ± SE. * *p* < 0.05, ** *p* < 0.01, *** *p* < 0.001, and **** *p*  <  0.0001; ns = not significant.

**Figure 11:**
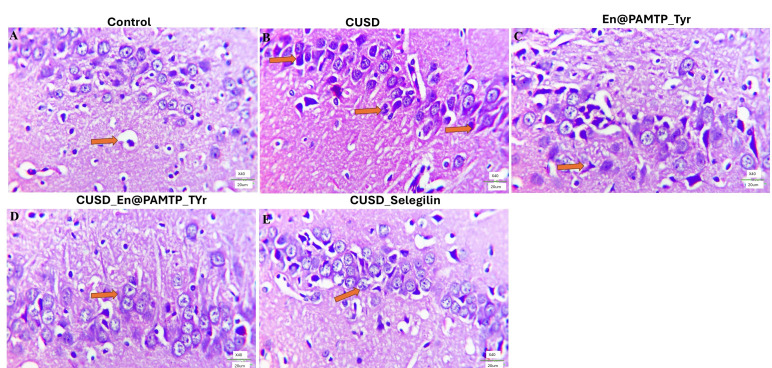
Photomicrograph of rat hippocampal CA1 stained with hematoxylin and eosin. These photomicrographs (magnification 40×, scale bar = 20 μm) show neuronal morphology in (**A**) control, (**B**) CUSD, (**C**) En@PAMTP_Tyr, (**D**) CUSD_ En@PAMTP_Tyr, and (**E**) CUSD_selegiline-treated group. Neuronal degeneration, shrinkage, and pyknotic nuclei (red arrows).

**Scheme 1:**
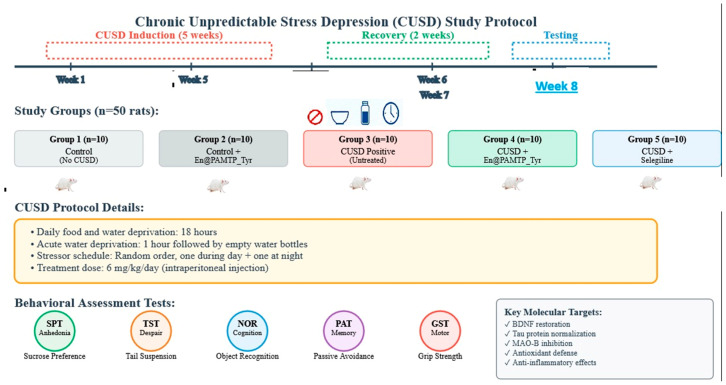
Experimental design diagram of the induction of chronic unpredictable stress-like depression and treatment with PAMTP.

**Scheme 2:**
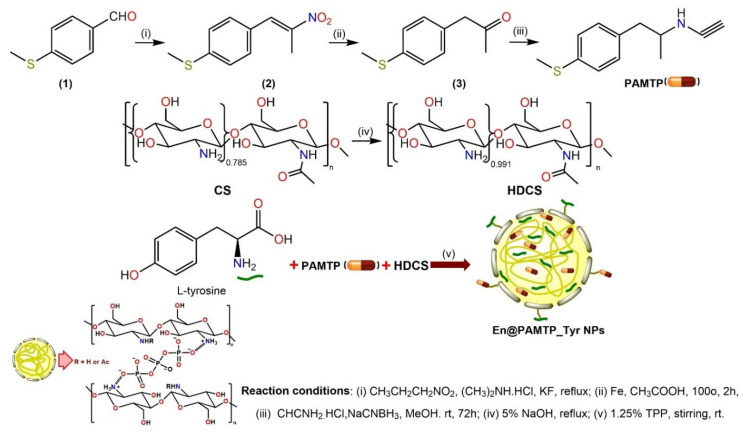
Synthesis of 2-N-Propargylamino-1-(4-methylthiophenyl)propane (PAMTP), highly deacetylated chitosan (HDCS), and PAMTP/Tyr-loaded chitosan nanoparticles (En@PAMTP_Tyr).

**Table 1:** Output results of the best conformers obtained from molecular docking simulations between PAMTP and selegiline against monoamine oxidase (ID: 3PO7) and acetylcholinesterase (ID: 5FPQ) receptors.

Parameter	PAMTP With Monoamine Oxidase ID: 3PO7	Selegiline With Monoamine Oxidase ID: 3PO7
Free energy of binding (kcal/mol).	−6.23	−5.96
Inhibition constant, Ki (µM).	26.98	42.75
Ref. RMSD	127.36	126.36
Hydrogen bonding	LIGH: TYR435(O), 2.2 A^o^.	LIGH: GLY434(O), 2.2 A^o^.
**Parameter**	**PAMTP with acetylcholinesterase, ID: 5FPQ.**	**Selegiline with acetylcholinesterase, ID: 5FPQ.**
Free energy of binding (kcal/mol).	−3.75	−3.54
Inhibition constant, Ki (mM).	1.8	2.55
Ref. RMSD	58.72	59.0
Hydrogen bonding	LIGH: ASP306(O), 2.1 A^o^.	None.

## Data Availability

The original contributions presented in this study are included in the article/Supplementary Materials. Further inquiries can be directed to the corresponding author.

## Supplementary Materials

The following supporting information can be downloaded at: https://www.mdpi.com/article/10.3390/biom16060871/s1, 1. Experimental part: 1.1. Materials; 1.2. Instrumentations; 1.3. Characterization of 2-N-Propargylamino-1-(4-methylthiophenyl)propane (PAMTP). Figure S1: ATR-IR spectrum of PAMTP; Figure S2: ^1^H NMR spectrum of PAMTP; Figure S3: UV-Vis spectrum of the native PAMTP; Figure S4: UV-Vis spectrum of the native Tyr; Figure S5: UV-Vis spectrum of the PAMTP after exposure to the harsh conditions (1 M solution of HCl at 95 °C for 30 min); Figure S6: UV-Vis spectrum of the Tyr after exposure to the harsh conditions (1 M solution of HCl at 95 °C for 30 min); Figure S7: EDX mapping images of the En@PAMTP_Tyr nanoformulation appear in the upper left and lower positions. The En@PAMTP_Tyr SEM image is shown in the upper-right corner; Table S1: fifty conformations detected from the molecular docking processes between 2-N-Propargylamino-1-(4-methylthiophenyl) propane, PAMTP and Selegiline against monoamine oxidase receptor, ID: 3PO7; Table S2: fifty conformations detected from the molecular docking processes between 2-N-Propargylamino-1-(4-methylthiophenyl)propane, PAMTP and Selegiline against acetylcholinesterase receptor, ID: 5FPQ.

## Abbreviations

The following abbreviations are used in this manuscript:

Abbreviation	Definition
AChE	Acetylcholinesterase
AD	Alzheimer’s Disease
ANOVA	Analysis of Variance
ARRIVE	Animal Research: Reporting of In Vivo Experiments
ATCI	Acetylthiocholine Iodide
BBB	Blood–Brain Barrier
BACE1	β-Secretase
BDNF	Brain-Derived Neurotrophic Factor
BSA	Bovine Serum Albumin
CA1	Cornu Ammonis area 1 (of the hippocampus)
CNS	Central Nervous System
CS	Chitosan
CUS	Chronic Unpredictable Stress
CUSD	Chronic Unpredictable Stress Depression-like
DAB	3,3′-Diaminobenzidine
DI	Discrimination Index
DLS	Dynamic Light Scattering
DTNB	5,5′-Dithiobis-(2-nitrobenzoic acid)
EDTA	Ethylenediaminetetraacetic Acid
EDX	Energy-Dispersive X-ray Spectroscopy
EE	Encapsulation Efficiency
ELISA	Enzyme-Linked Immunosorbent Assay
En@PAMTP_Tyr	Chitosan Nanoparticles Co-encapsulating PAMTP and L-Tyrosine
En@Empty	Blank Chitosan Nanoparticles (without PAMTP/Tyr)
ESM	Electronic Supplementary Material
FAD	Flavin Adenine Dinucleotide
FTIR	Fourier-Transform Infrared Spectroscopy
GSH	Glutathione
H&E	Hematoxylin and Eosin
HCl	Hydrochloric Acid
HDCS	Highly Deacetylated Chitosan
HRP	Horseradish Peroxidase
IAEC	Institutional Animal Ethics Committee
IL-10	Interleukin-10
IP	Intraperitoneal
LAT1	L-Type Amino Acid Transporter 1
LC	Loading Capacity
L-DOPA	L-3,4-Dihydroxyphenylalanine
LTP	Long-Term Potentiation
MAO	Monoamine Oxidase
MAO-A	Monoamine Oxidase A
MAO-B	Monoamine Oxidase B
MAPK/ERK	Mitogen-Activated Protein Kinase/Extracellular Signal-Regulated Kinase
MDD	Major Depressive Disorder
NADH	Nicotinamide Adenine Dinucleotide (Reduced)
NaOH	Sodium Hydroxide
NOR	Novel Object Recognition
PAMTP	2-N-Propargylamino-1-(4-methylthiophenyl) propane
PBS	Phosphate-Buffered Saline
PBST	Phosphate-Buffered Saline with Tween
PD	Parkinson’s Disease
PI3K/Akt	Phosphoinositide 3-Kinase/Protein Kinase B
ROS	Reactive Oxygen Species
SEM	Scanning Electron Microscopy
SOD	Superoxide Dismutase
TEM	Transmission Electron Microscopy
TMB	3,3′,5,5′-Tetramethylbenzidine
TNB	5-Thio-2-nitrobenzoic acid
TNF-α	Tumor Necrosis Factor-alpha
TP	Tissue Protein
TPP	Sodium Tripolyphosphate
Tris	Tris(hydroxymethyl)aminomethane
TST	Tail Suspension Test
Tyr	L-Tyrosine
UV-Vis	Ultraviolet-Visible Spectroscopy
