# Supplementary material for: Chitosan Nanoparticles Co-Encapsulating Selegiline Analogue and L-Tyrosine Mitigate Depression-Related Pathology and Cognitive Decline in Rats
Source: Biomolecules. 2026 Jun 14;16(6):871. doi: 10.3390/biom16060871 (PMC13297493; doi:10.3390/biom16060871)
Supplement: Supplementary file 1 [file biomolecules-16-00871-s001.zip › biomolecules-4323237-supplementary.pdf]

Chitosan Nanoparticles Co-Encapsulating Selegiline Analogue and L-Tyrosine Mitigate Depression-Related Pathology and Cognitive Decline in Rats

Wesam Abd El-Fattah , Ahlem Guesmi * , Naoufel Ben Hamadi , Khulud M. Alshehri ,
Ehab Mohamed Abdella [3,4], Rehab R. Mohamed [5], Reda F. M. Elshaarawy [6,*] and Hani S. Hafez [7]

[1] Department of Chemistry, College of Science, Imam Mohammad Ibn Saud Islamic University (IMSIU), P.O. Box 5701, Riyadh 11623, Saudi Arabia; wabdulfatah@imamu.edu.sa (W.A.E.-F.); nabenhamadi@imamu.edu.sa (N.B.H.)
[2] Department of Biology, Al-Baha University, Al Baha 65431, Saudi Arabia; kalshehri@bu.edu.sa
[3] Zoology Department, Faculty of Science, Beni Suef University, Beni-Suef 62521, Egypt; immohammad@bu.edu.sa
[4] Biology Department, Faculty of Science, Al-Baha University, Al-Baha 65779, Saudi Arabia
[5] Zoology Department, Science Faculty, Fayoum University, Fayoum 63514, Egypt; rrm01@fayoum.edu.eg
[6] Department of Chemistry, Faculty of Science, Suez University, Suez 43533, Egypt
[7] Department of Zoology, Faculty of Science, Suez University, Suez 43533, Egypt; hani.hafez@suezuniv.edu.eg

* Correspondence: amalkasme@imamu.edu.sa (A.G.); reda.elshaarawy@suezuniv.edu.eg (R.F.M.E.); Tel.: +002-01017377216 (R.F.M.E.)

1. Experimental part

1.1. Materials

CA was purchased from Aoxing Biotechnology. N-hydroxysuccinimide (NHS) was purchased from Aladdin (China). L-tyrosine. The N-(3-dimethylaminopropy)-N-ethylcarbodiimide hydrochloride (EDC), and 1-hydroxybenzotriazole (HOBT) were purchased from Meryer Chemical Technology Co. Ltd. (Shanghai), and Genipin was purchased from Zhixin Biology.

1.2. Instrumentations

Melting points were determined using a BUCHI B-540 (BÜCHI, Switzerland) melting point instrument. Measurements were taken in open glass capillaries and reported without corrections. A BRUKER Tensor-37 FTIR spectrophotometer (BRUKER, USA) was employed to measure FTIR spectra. The measurements were performed in the range of 400-4000 cm^{-1} using KBr discs, with a resolution of 2 cm^{-1}. The absorption bands were categorized as vs. (very strong), s (strong), w (weak), m (medium), sh (sharp), and br (broad). The acquisition of NMR spectra was conducted using two different instruments: a Bruker Avance DRX200, which operates at 200 MHz for ^{1}H-NMR, and a Bruker Avance DRX500, operating at 125 MHz for ^{13}C-NMR. For calibration, the residual proton solvent signals were utilized.

DMSO-d6 displayed ^{1}H NMR and 13C NMR signals at 2.52 ppm and 39.5 ppm, respectively. Alternatively, $CDCl_3$ exhibited signals at 7.26 ppm for ^{1}H NMR and 77.16 ppm for 13C NMR. TMS was used as the reference standard, with δ assigned a value of 0.00 ppm, for both 1H and ^{13}C measurements. The peak patterns were denoted as follows: singlet (s), doublet (d), triplet (t), quartet (q), or multiplet (m) to indicate signal multiplicities. A UHR-QTOF maXis 4 G (Bruker Daltonics) and a BRUKER Ultraflex MALDI-TOF system were utilized for electrospray ionization mass spectrometry (ESI-MS) analysis. The measurements were conducted in linear mode for positive ions. The MALDI-TOF instrument featured a nitrogen laser with a wavelength of 337 nm, operating at a 10 Hz frequency. In ESI-MS, the 2+ charge state of ions was evidenced by the 0.5 m/z separation between successive isotope peaks (x, x + 1, x + 2).

The textural and physical features of the new material were visualized using scanning electron microscope (SEM) from Hitachi 3400 N (Hitachi, Japan). The samples for SEM observations were observed without any metal coating. A high-resolution transmission electron microscope (HR-TEM, Tecnai G20, FEI, Netherland) was used for imaging, crystal structure revelation, and elemental analysis.

The UV analysis was performed using a UV–Visible spectrophotometer (Shimadzu UV-2600) (Shimadzu, Japan). The particle size distribution was determined using the Dynamic Light Scattering (DLS) technique by Zetasizer Nano ZS90 (Malvern Instrument Ltd.). The experiment was carried out at room temperature using nanoparticles dissolved in water as the solvent.

1.3. Characterization of of 2-N-Propargylamino-1-(4-methylthiophenyl)propane (PAMTP)

IR (ATR, *ν* cm^{-1}) 3310, 2108, 1601 (**Figure S1**). ^{1}H NMR δ ($CDCl_3$, 200 Hz) 17.50 (dd, *J* = 7.5, 1.7 Hz, 2H), 7.41 (dd, *J* = 7.7, 1.7 Hz, 2H), 3.54 – 3.30 (m, 1H), 3.04 (s, 1H), 2.90 (d, *J* = 7.0 Hz, 2H), 2.45 (s, 3H), 1.64, 1.30 (d, *J* = 6.9 Hz, 3H) (**Figure S2**). **HCl salt**: Colourless solid crystals, mp 176–178 ^{o}C. Anal. Calculated for $C_{13}H1_{8}ClNS$: C, 61.04; H, 7.09; N, 5.48; S, 12.54. Found: C, 60.96; H, 7.11; N, 5.42; S, 12.49%.

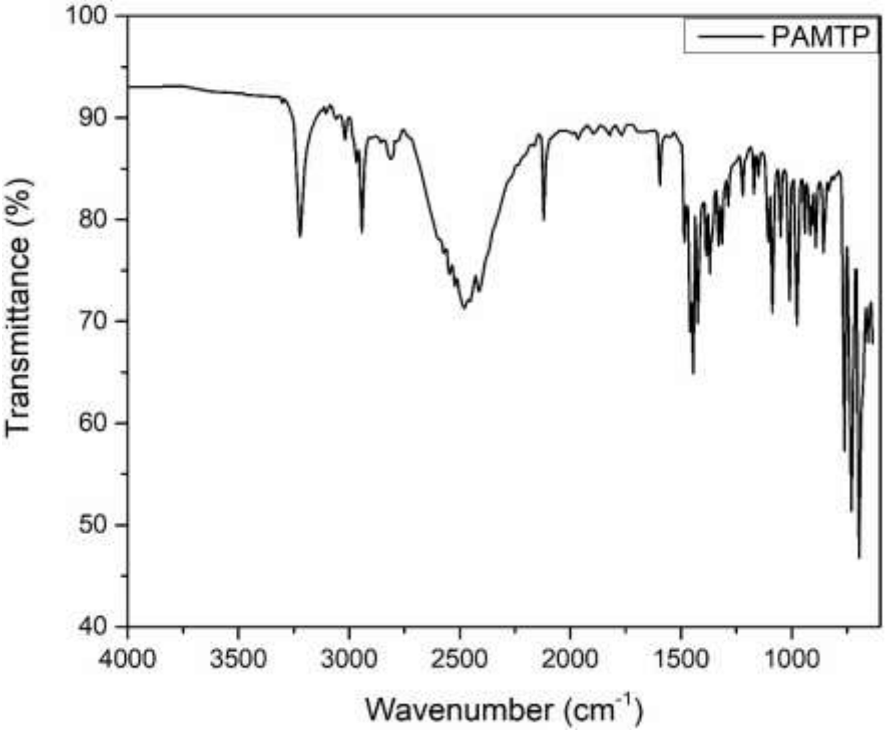

Figure S1: ATR-IR spectrum of **PAMTP**

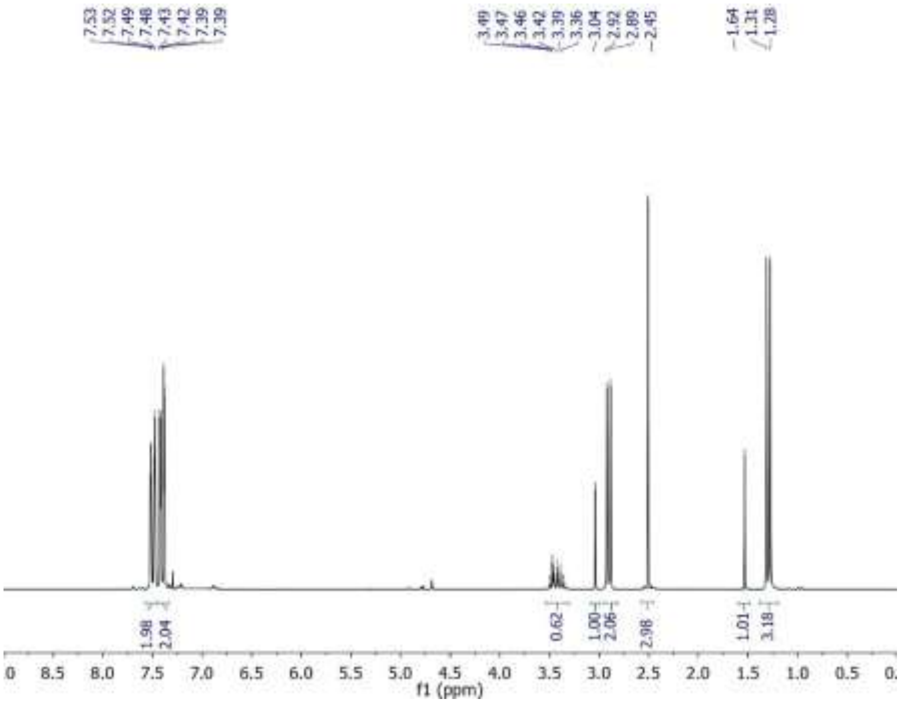

Figure S2: ^{1}H NMR spectrum of **PAMTP**

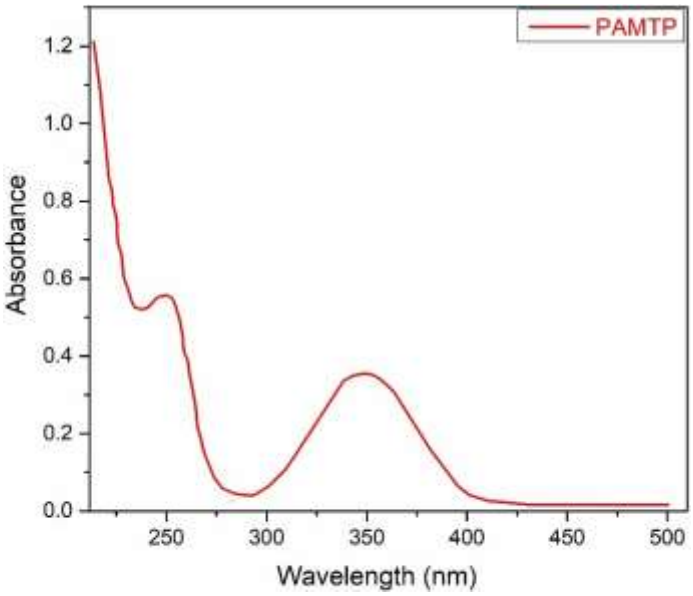

Figure S3: UV-Vis spectrum of the native PAMTP

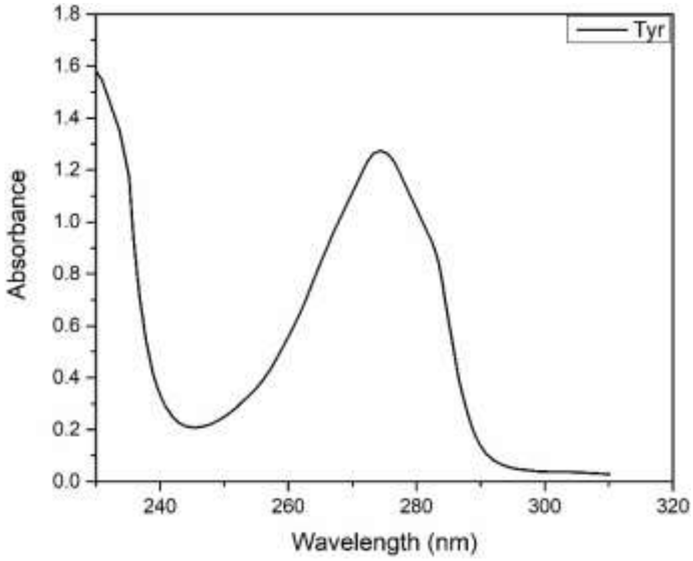

Figure S4: UV-Vis spectrum of the native Tyr

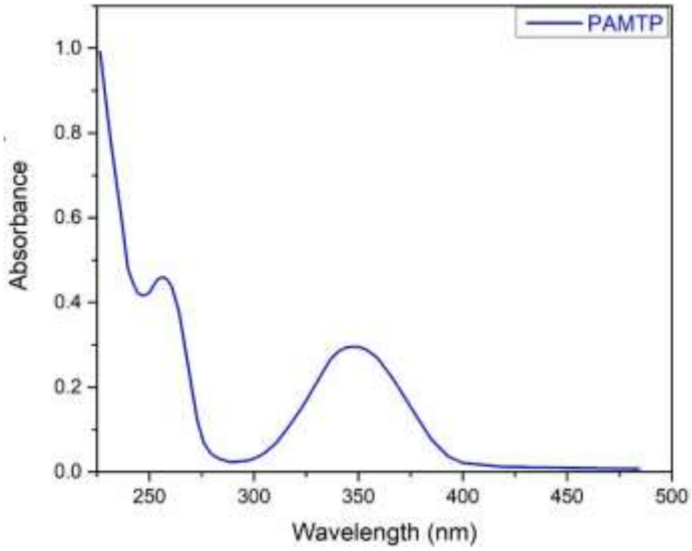

Figure S5: UV-Vis spectrum of the PAMTP after exposure to the harsh conditions (1 M solution of HCl at 95 °C for 30 min)

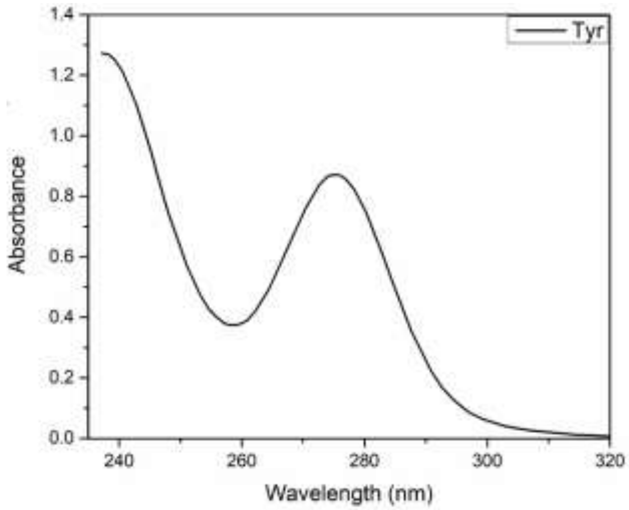

Figure S6: UV-Vis spectrum of the Tyr after exposure to the harsh conditions (1 M solution of HCl at 95 °C for 30 min)

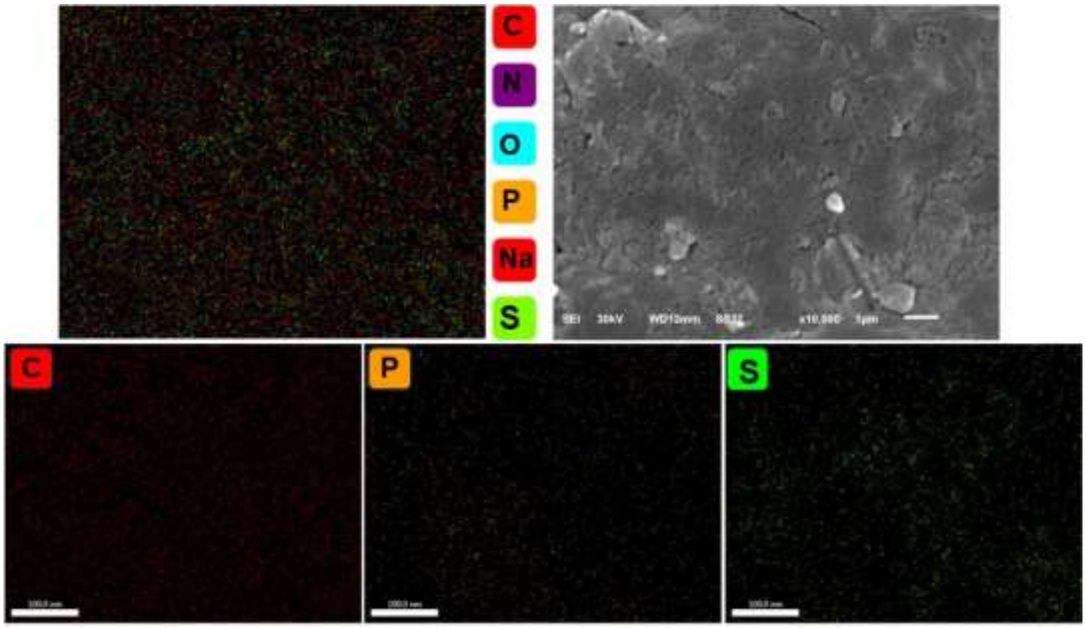

Figure S7: EDX mapping images of the En@PAMTP_Tyr nanoformulation appear in the upper left and lower positions. The En@PAMTP_Tyr SEM image is shown in the upper-right corner.

Molecular docking analysis

Table S1: fifty conformations detected from the molecular docking processes between 2-N-Propargylamino-1-(4-methylthiophenyl) propane, PAMTP and Selegiline against monoamine oxidase receptor, ID: 3PO7.

Conformer's No.	PAMTP With monoamine oxidase, ID: 3PO7.		Selegiline With monoamine oxidase, ID: 3PO7.	
	Free energy of binding (Kcal/mol).	Inhibition constant, Ki.	Free energy of binding (Kcal/mol).	Inhibition constant, Ki.
1	-6.15	30.84 uM	-5.96	42.75 uM
2	-6.18	29.60 uM	-5.75	61.43 uM
3	-6.10	33.62 uM	-5.94	44.15 uM
4	-6.09	34.46 uM	-5.70	66.22 uM
5	-5.72	63.69 uM	-5.83	53.71 uM
6	-5.97	42.14 uM	-5.67	69.63 uM
7	-6.18	29.62 uM	-5.58	80.83 uM
8	-6.17	29.85 uM	-5.67	69.29 uM
9	-6.12	32.74 uM	-5.57	82.98 uM
10	-5.98	41.18 uM	-5.70	66.12 uM
11	-6.09	34.39 uM	-5.71	64.81 uM
12	-5.78	58.44 uM	-5.76	60.03 uM
13	-5.55	85.97 uM	-5.54	86.26 uM
14	-6.09	34.07 uM	-5.70	66.80 uM
15	-5.98	41.05 uM	-5.67	69.49 uM
16	-5.71	65.53 uM	-5.89	48.05 uM
17	-6.14	31.49 uM	-5.63	74.36 uM
18	-6.10	33.69 uM	-5.65	72.28 uM
19	-5.72	63.90 uM	-5.67	70.32 uM
20	-5.73	63.38 uM	-5.87	49.78 uM
21	-6.14	31.58 uM	-5.63	74.84 uM
22	-6.10	33.71 uM	-5.63	74.98 uM
23	-5.68	69.03 uM	-5.63	74.64 uM
24	-6.13	32.03 uM	-5.67	69.44 uM
25	-6.16	30.71 uM	-5.71	65.18 uM
26	-6.14	31.38 uM	-5.65	72.31 uM
27	-6.14	31.54 uM	-5.63	74.64 uM
28	-6.23	26.98 uM	-5.93	44.93 uM
29	-5.86	50.37 uM	-5.70	66.58 uM
30	-5.91	46.43 uM	-5.65	72.20 uM
31	-5.85	51.66 uM	-5.81	55.19 uM
32	-5.70	66.87 uM	-5.95	43.34 uM
33	-5.88	48.74 uM	-5.74	61.86 uM
34	-6.11	33.27 uM	-5.81	54.71 uM
35	-6.18	29.32 uM	-5.91	46.87 uM
36	-6.18	29.65 uM	-5.70	66.05 uM

37	-6.22	27.69 uM	-5.70	66.63 uM
38	-6.09	34.30 uM	-5.68	68.54 uM
39	-5.53	88.91 uM	-5.74	62.52 uM
40	-5.70	65.88 uM	-5.88	49.12 uM
41	-6.16	30.74 uM	-5.66	71.57 uM
42	-6.23	27.24 uM	-5.68	68.12 uM
43	-5.81	55.47 uM	-5.71	65.11 uM
44	-5.73	63.44 uM	-5.88	48.58 uM
45	-5.82	53.89 uM	-5.66	71.28 uM
46	-6.09	34.23 uM	-5.68	68.62 uM
47	-6.21	27.82 uM	-5.65	71.70 uM
48	-5.93	44.82 uM	-5.73	63.50 uM
49	-5.69	67.72 uM	-5.95	43.41 uM
50	-6.09	34.45 uM	-5.73	63.44 uM

Table S2: fifty conformations detected from the molecular docking processes between 2-N-Propargylamino-1-(4-methylthiophenyl)propane, PAMTP and Selegiline against acetylcholinesterase receptor, ID: 5FPQ.

Conformer's No.	PAMTP With acetylcholinesterase, ID: 5FPQ.		Selegiline With acetylcholinesterase, ID: 5FPQ.	
	Free energy of binding (Kcal/mol).	Inhibition constant, Ki.	Free energy of binding (Kcal/mol).	Inhibition constant, Ki.
1	-3.10	5.36 mM	-3.00	6.30 mM
2	-3.61	2.25 mM	-3.27	3.99 mM
3	-3.00	6.36 mM	-3.33	3.60 mM
4	-3.45	2.97 mM	-3.31	3.73 mM
5	-3.16	4.84 mM	-3.09	5.40 mM
6	-3.32	3.68 mM	-3.25	4.18 mM
7	-3.27	4.03 mM	-3.07	5.66 mM
8	-3.15	4.89 mM	-3.07	5.59 mM
9	-3.27	4.03 mM	-3.05	5.85 mM
10	-3.28	3.95 mM	-3.44	3.02 mM
11	-3.71	1.90 mM	-2.94	7.01 mM
12	-3.13	5.09 mM	-3.06	5.73 mM
13	-3.04	5.88 mM	-3.13	5.12 mM
14	-3.01	6.17 mM	-3.26	4.09 mM
15	-3.48	2.80 mM	-2.94	7.00 mM
16	-3.43	3.07 mM	-3.50	2.74 mM
17	-3.00	6.32 mM	-2.92	7.28 mM
18	-3.42	3.13 mM	-2.95	6.91 mM
19	-3.75	1.80 mM	-3.48	2.82 mM
20	-2.82	8.54 mM	-3.38	3.33 mM
21	-3.31	3.77 mM	-3.51	2.68 mM
22	-3.57	2.42 mM	-2.93	7.11 mM

23	-3.15	4.92 mM	-3.40	3.19 mM
24	-2.94	6.98 mM	-2.92	7.26 mM
25	-3.56	2.46 mM	-3.02	6.12 mM
26	-3.14	4.98 mM	-3.16	4.82 mM
27	-3.13	5.10 mM	-3.32	3.70 mM
28	-2.72	10.19 mM	-3.10	5.37 mM
29	-2.83	8.46 mM	-2.93	7.15 mM
30	-3.11	5.24 mM	-2.77	9.40 mM
31	-3.15	4.90 mM	-3.14	5.03 mM
32	-2.92	7.23 mM	-3.11	5.24 mM
33	-2.82	8.55 mM	-2.97	6.69 mM
34	-3.18	4.69 mM	-3.54	2.55 mM
35	-3.02	6.14 mM	-3.09	5.40 mM
36	-3.10	5.36 mM	-3.10	5.38 mM
37	-3.03	5.97 mM	-2.97	6.66 mM
38	-3.00	6.31 mM	-2.92	7.22 mM
39	-3.57	2.40 mM	-3.21	4.44 mM
40	-2.97	6.68 mM	-3.28	3.94 mM
41	-3.18	4.64 mM	-3.02	6.11 mM
42	-2.99	6.45 mM	-2.87	7.85 mM
43	-2.92	7.26 mM	-3.27	4.02 mM
44	-3.33	3.64 mM	-3.38	3.32 mM
45	-3.11	5.24 mM	-3.33	3.63 mM
46	-3.39	3.28 mM	-2.84	8.28 mM
47	-2.68	10.83 mM	-2.91	7.36 mM
48	-2.91	7.35 mM	-3.48	2.84 mM
49	-3.43	3.04 mM	-3.39	3.28 mM
50	-3.53	2.57 mM	-3.53	2.57 mM
